# Fat-Dachsous planar polarity function requires two distinct heterophilic cadherin-cadherin binding interactions

**DOI:** 10.1016/j.celrep.2024.114722

**Published:** 2024-09-19

**Authors:** Helen Strutt, Dipak Meshram, Elizabeth Manning, Amritha Chemmenchery Kokkam Madathil, David Strutt

**Affiliations:** 1 School of Biosciences, University of Sheffield, Sheffield S10 2TN, UK

**Keywords:** *Drosophila*, development, wing patterning, planar polarity, planar cell polarity, PCP, Fat, Dachsous, Fat-Dachsous pathway, cadherin

## Abstract

Fat and Dachsous are evolutionarily conserved atypical cadherins that regulate polarized cell behaviors. In the *Drosophila* wing, they interact heterophilically between neighboring cells, localize asymmetrically to opposite cell ends, and control wing shape by regulating oriented cell rearrangements and divisions. Fat and Dachsous have 34 and 27 cadherin repeats, respectively, and previous work has identified *trans* interactions between their first four cadherin repeats. Here, we identify a second heterophilic binding site in their C-terminal cadherin repeats and show the conservation of this binding site in human Fat4 and Dachsous1. We provide evidence that both N- and C-terminal binding sites regulate the stability of Fat-Dachsous binding interactions and show that the N-terminal binding sites are partly dispensable for Fat-Dachsous function *in vivo*. Finally, we provide *in vivo* confirmation that the N-terminal repeats interact in an anti-parallel manner. We propose that multiple binding sites promote the clustering of Fat and Dachsous into a lattice-like array.

## Highlights

• N- and C-terminal cadherin binding sites mediate Fat and Dachsous *trans* interactions
• N- and C-terminal cadherin sites act redundantly to regulate the stability of binding
• N-terminal cadherin binding is not essential for Fat-Dachsous function *in vivo*
• N-terminal cadherin repeats of Fat and Dachsous interact in a head-to-tail manner

## Graphical abstract

**Figure:**
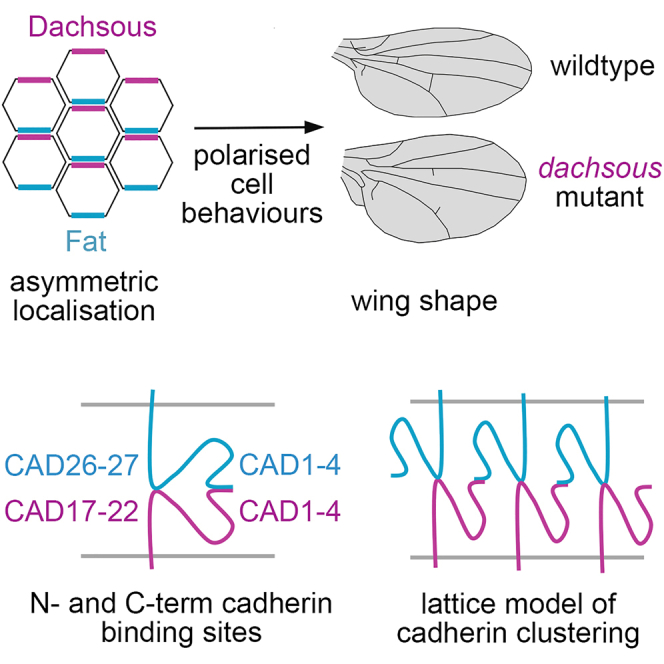

## Teaser

Fat and Dachsous interact in neighboring cells via their cadherin repeats to regulate cell behaviors and tissue shape. Strutt et al. show that interactions between two distinct cadherin regions in each molecule regulate the stability of Fat-Dachsous binding and modulate Fat-Dachsous function in the developing fly wing.

## Introduction

The Fat-Dachsous (Ft-Ds) pathway is conserved across the animal kingdom,^1^^,^^2^ regulating tissue morphogenesis and growth, with loss of function leading to congenital birth defects and human disease.^2^^,^^3^ A major role is to specify planar polarity in epithelia, such that cells adopt a common polarity in the plane of the tissue, in turn controlling polarized cell behaviors.

**Figure 1:**
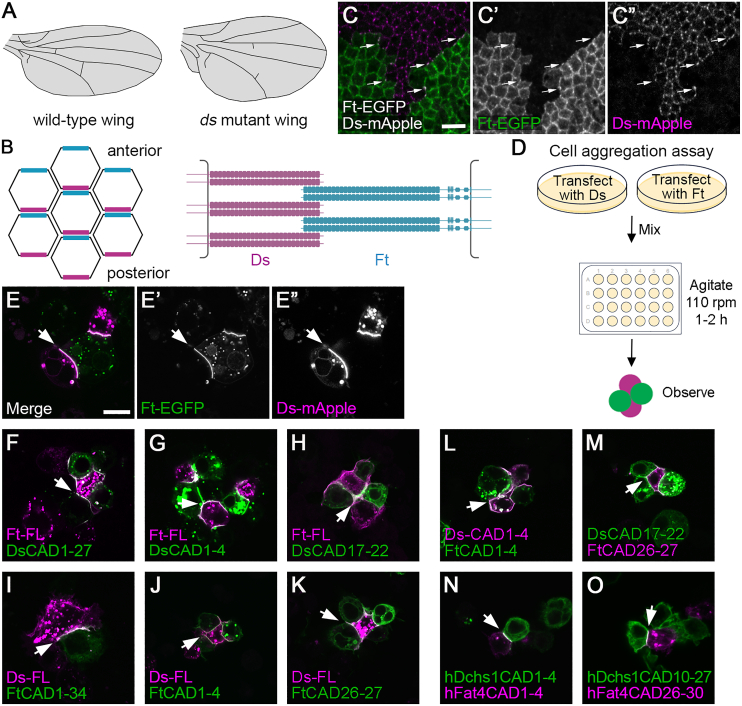
Identification of N-terminal and C-terminal heterophilic binding sites in the CAD repeats of Ds and Ft (A) Diagram of a wild-type wing (left) and a *ds* mutant wing (right). (B) Diagrams illustrating the subcellular localizations (left) and structures (right) of Ds (purple) and Ft (cyan). In the posterior pupal wing, Ds localizes to posterior cell edges and Ft to anterior cell edges. Ds has 27 CAD repeats, while Ft has 34 CAD repeats and more C-terminal epidermal growth factor (EGF) and LamG domains. DsCAD1–4 interact heterophilically with FtCAD1–4. (C) Third-instar wing imaginal disc showing a clone of cells expressing Ft-EGFP (green) next to cells expressing Ds-mApple (magenta). Arrows indicate junctional puncta where Ft and Ds in neighboring cells are concentrated and interact. Scale bar, 5 μm. (D) Diagram illustrating the S2 cell aggregation assay. (E) S2 cell aggregation assay where cells transfected with Ft-mEGFP (green) were mixed with cells expressing Ds-mApple (magenta). Arrows point to interfaces between Ds- and Ft-expressing cells. Scale bar, 10 μm. (F–K) Aggregation experiments between cells expressing full-length (FL) Ft-mApple (F–H, magenta) or FL Ds-mApple (I–K, magenta) and cells expressing cell surface CAD repeats tagged with EGFP, as indicated (green). (L–O) Aggregation experiments between cells expressing cell surface CAD repeats, tagged with EGFP (green) or HA (magenta). See also Figure S1.

During *Drosophila* wing development, loss of Ft-Ds planar polarity activity results in wings becoming rounder (Figure 1A),^4^^,^^5^^,^^6^^,^^7^ apparently due to defects in oriented cell divisions and rearrangements.^6^^,^^8^^,^^9^ Ft-Ds planar polarity also regulates the growth/size of the wing via Hippo-Warts signaling.^2^^,^^3^

Ft and Ds are protocadherin superfamily members. They specify planar polarity at the cellular level by localizing asymmetrically to opposite sides of cells,^10^^,^^11^^,^^12^ where they interact heterophilically via their cadherin (CAD) repeats, forming intercellular contacts (Figures 1B and 1C).^13^^,^^14^^,^^15^ The vertebrate homologs Fat4 and Dachsous1 (Dchs1) similarly bind heterophilically in *trans*^16^^,^^17^^,^^18^ and can also planar polarize.^18^ Heterophilic binding is regulated by the phosphorylation of specific CAD repeats by the Golgi kinase Four-jointed (Fj),^19^^,^^20^^,^^21^^,^^22^ converting Ds/Fj expression gradients into Ft-Ds asymmetric localization.^23^

Ft and Ds contain 34 and 27 CAD repeats, respectively (Figure 1B),^4^^,^^5^ but the function of most of the repeats is unknown. Previous work has suggested that the first four CAD repeats are sufficient for Ft-Ds binding,^17^^,^^21^^,^^22^^,^^24^ and phosphorylation of a subset of the first ten CAD repeats by Fj can modulate Ft-Ds binding.^21^^,^^22^^,^^23^ However, whether the other CAD repeats mediate heterophilic *trans* interactions has not been investigated. Alternatively, some CAD repeats could have a role in stabilizing arrays of Ft-Ds heterodimers via homophilic *cis* interactions, as seen for E-cadherin and protocadherins.^25^^,^^26^^,^^27^^,^^28^ Furthermore, it is unclear how the large extracellular domains of Ft and Ds fit into the intercellular space at adherens junctions. Evidence from purified ectodomains reveals that Ft and Ds form "kinks" between specific CAD repeats that lack calcium binding motifs, and this may assist in their packing.^17^

In this work, we use a tissue culture assay to carry out a comprehensive analysis of the CAD domains required for heterophilic Ft-Ds *trans* interactions. In addition to the known binding between the first four CAD domains, we identify conserved C-terminal CAD binding sites in both Ft and Ds that mediate heterophilic *trans* interactions. Importantly, we demonstrate the physiological relevance of the second interaction sites in wing development *in vivo*. Finally, we show that in cultured cells, FtCAD1–4 and DsCAD1–4 interact in an antiparallel "head-to-tail" manner, consistent with a recent *in vitro* structure for mammalian Fat4 and Dchs1.^24^

## Results

### Ft and Ds CAD regions interact in *trans* via both N-terminal and C-terminal binding sites

We used a cell aggregation assay to dissect the CAD domains involved in heterophilic *trans* interactions between Ft and Ds (Figure 1D). S2 cells expressing full-length Ft-mEGFP were mixed with cells expressing full-length Ds-mApple. As previously shown,^15^^,^^21^^,^^29^ Ft and Ds expressing cells aggregated, and Ft and Ds co-localized at sites of cell contacts, consistent with the formation of heterophilic *trans* interactions (Figure 1E).

To identify the minimal CAD domains required for heterophilic *trans* interactions, the entire CAD regions of Ds or Ft were inserted in a heterologous construct with a downstream transmembrane (TM) domain from the unrelated CD2 cell surface protein. Cells expressing Ds[CAD1–27]-EGFP aggregated with cells expressing full-length Ft-mApple, and cells expressing Ft[CAD1–34]-EGFP aggregated with cells expressing full-length Ds-mApple. In both cases, there was a co-localization of EGFP and mApple at the cell interfaces (Figures 1F–1I).

Ds and Ft constructs containing subsets of CAD repeats were then generated (Table S1) and tested for their ability to bind to the other full-length molecules in neighboring cells (Figures S1A and S1B). This revealed two sets of CAD repeats in each molecule that were sufficient for binding. DsCAD1–4 or DsCAD17–22 were sufficient to bind full-length Ft (Figures 1G, 1H, and S1A), and FtCAD1–4 or FtCAD26–27 were sufficient to bind full-length Ds (Figures 1J, 1K, and S1B). The N-terminal binding sites are consistent with previous reports.^17^^,^^21^^,^^22^^,^^24^

We then tested whether the CAD domains we identified could interact in *trans* in our assay. We confirmed the interaction between DsCAD1–4 and FtCAD1–4 (Figures 1L, S1C, and S1E) and found that DsCAD17–22 interacted in *trans* with FtCAD26–27 (Figures 1M, S1D, and S1F). We also showed that these binding sites were exclusive: DsCAD1–4 did not bind FtCAD26–27, and FtCAD1–4 did not bind DsCAD17–22 (Figures S1C–S1F).

Finally, we examined whether the N- and C-terminal binding sites were conserved in human Dchs1 (hDchs1) and human Fat4 (hFat4). As expected, hDchs1CAD1–4 interacted with hFat4CAD1–4 (Figure 1N). hDchs1CAD17–22 did not appear to reach the cell surface in S2 cells, nor did the slightly larger hDchs1CAD15–24. However, hDchs1CAD10–27 interacted in *trans* with hFat4CAD26–30 (Figure 1O), consistent with the conservation of the C-terminal binding site.

### Head-to-tail binding of FtCAD1–4 and DsCAD1–4

**Figure 2:**
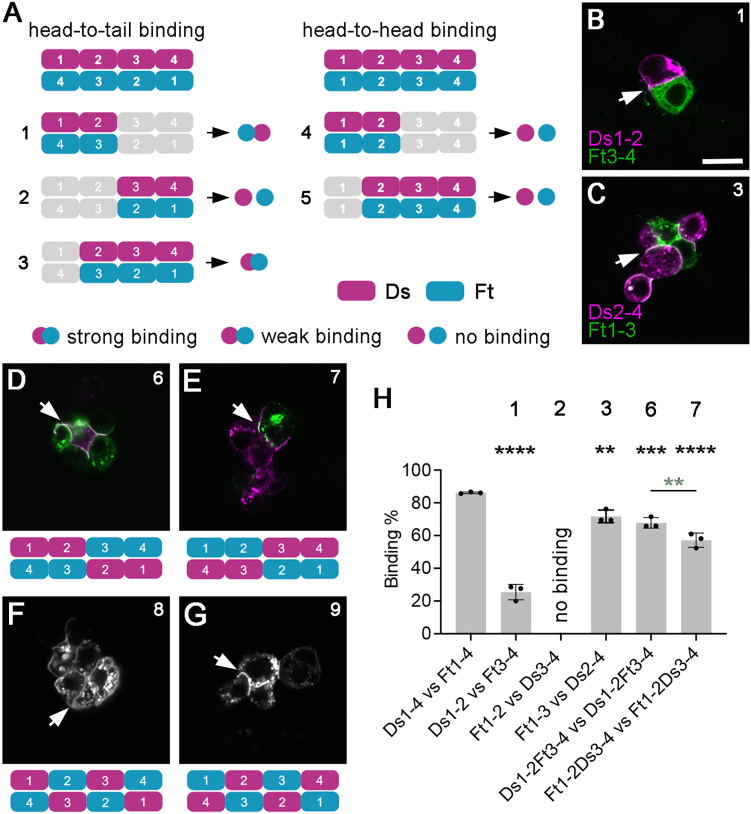
DsCAD1–4 and FtCAD1–4 interact head to tail (A) Diagrams illustrating binding of DsCAD1–4 (purple) and FtCAD1–4 (cyan) in a head-to-tail (left) or head-to-head (right) configuration. Diagrams on the bottom show the results of aggregation experiments to test the binding between cells expressing subsets of cell surface DsCAD1–4 or FtCAD1–4 as indicated. Binding was scored as strong (clear interface present), weak (cells touch but no clear interface present), or no binding. Ft constructs were tagged with EGFP and Ds constructs with HA or mApple. (B–G) Aggregation experiments between cells expressing cell surface CAD repeats. (B) DsCAD1–2-HA with FtCAD3–4-EGFP, experiment 1. (C) DsCAD2–4-mApple with FtCAD3–4-EGFP, experiment 3. (D) DsCAD1–2-FtCAD3–4 tagged with EGFP and mApple, experiment 6. (E) FtCAD1–2-DsCAD3–4 tagged with EGFP and HA, experiment 7. (F) DsCAD1-FtCAD2-DsCAD3-FtCAD4 tagged with mApple, experiment 8. (G) FtCAD1-DsCAD2-FtCad3-DsCAD4 tagged with EGFP, experiment 9. Images show EGFP fluorescence (green in B–E, white in G) and mApple fluorescence or immunolabeling for HA (magenta in B–E, white in F). Arrows point to interfaces between Ds- and Ft-expressing cells. Scale bar, 10 μm. (H) Scoring of percentage of binding between cells in experiments 1, 2, 3, 6, and 7. Error bars are standard deviation (SD), *n* = 3. Samples were compared to DsCAD1–4 binding to FtCAD1–4 using ANOVA with Dunnett’s multiple comparisons test (black asterisks) or experiments 6 and 7 were compared using ANOVA with Šidák’s multiple comparisons test (gray asterisks). ^∗∗^*p* < 0.01, ^∗∗∗^*p* < 0.001, and ^∗∗∗∗^*p* ≤ 0.0001. See also Table S2.

Recent structural studies suggest that CAD1–4 of mammalian Fat4 and Dchs1 interact *in vitro* with a head-to-tail organization.^24^ We wanted to confirm this for fly Ft and Ds in our assay and also determine if fewer than four CAD domains were sufficient for binding. If FtCAD1–4 and DsCAD1–4 bind head to tail, then DsCAD1 would interact with FtCAD4, and so on (Figure 2A, top left). Alternatively, head-to-head binding would result in DsCAD1 interacting with FtCAD1, and so on (Figure 2A, top right).

We tested these two possibilities using subsets of CAD1–4. Interestingly, DsCAD1–2 was sufficient to bind FtCAD3–4 (Figures 2A, experiment 1, and 2B). DsCAD3–4 did not bind FtCAD1–2 (Figure 2A, experiment 2), but DsCAD2–4 bound FtCAD1–3 (Figures 2A, experiment 3, and 2C). This supports head-to-tail binding. Conversely, DsCAD1–2 did not bind FtCAD1–2, nor did DsCAD2–4 bind FtCAD2–4 (Figure 2A, experiments 4 and 5). Thus, these subsets of CAD domains do not bind head to head.

To further test for anti-parallel binding, chimaeras were made between various CAD repeats of Ds and Ft. A DsCAD1–2-FtCAD3–4 chimaera interacted with itself in *trans* (Figure 2D, experiment 6). Notably, aggregation efficiency was significantly increased compared to an isolated DsCAD1–2 interacting with an isolated FtCAD3–4 (Figure 2H, experiments 1 and 6; Table S2). In the converse experiment, as above, FtCAD1–2 did not interact with DsCAD3–4 (Figure 2A, experiment 2), but a FtCAD1–2-DsCAD3–4 chimaera could interact with itself (Figure 2E, experiment 7). Binding efficiency was lower than for DsCAD1–2-FtCAD3–4 (Figure 2H, experiments 6 and 7; Table S2).

Chimaeras consisting of alternating Ds and Ft CAD repeats also interacted together (Figures 2F and 2G). Thus, our data demonstrate anti-parallel binding in cell culture and show that all four CAD repeats of Ft and Ds contribute to the overall binding affinity, but DsCAD1–2 binding to FtCAD3–4 is stronger than DsCAD3–4 binding to FtCAD1–2.

### The N-terminal and C-terminal CAD binding sites both contribute to the stability of Ft-Ds interactions

**Figure 3:**
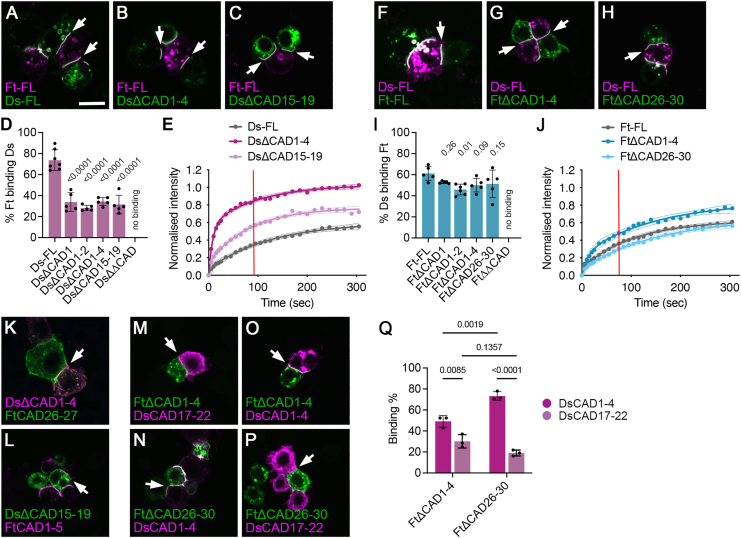
N-terminal and C-terminal CAD binding sites regulate the stability of Ft-Ds heterophilic interactions (A–C and F–H) Aggregation experiments between cells expressing (A–C) FL Ft-mApple and cells expressing Ds-mEGFP (A), Ds^ΔCAD1–4^-mEGFP (B), or Ds^ΔCAD15–19^-mEGFP (C) or (F–H) FL Ds-mApple and cells expressing Ft-mEGFP (F), Ft^ΔCAD1–4^-mEGFP (G), or Ft^ΔCAD26–30^-mEGFP (H). Arrows point to interfaces between Ds- and Ft-expressing cells. Scale bar, 10 μm. (D and I) Scoring of percentage of cells expressing Ft-mApple binding to cells expressing FL Ds-mEGFP or versions with CAD deletions (D) or cells expressing Ds-mApple binding to cells expressing FL Ft-mEGFP or versions with CAD deletions (I). Both CAD domains were deleted in Ds^ΔΔCAD^ and Ft^ΔΔCAD^. Error bars are SD, *n* = 5 for all samples except (D) Ds-FL, *n* = 7, and (I) FtΔCAD1–2, *n* = 6. Samples were compared to FL using ANOVA with Dunnett’s multiple comparisons test. (E and J) FRAP experiments measuring recovery of mEGFP fluorescence on interfaces between (E) cells expressing Ft-mApple and cells expressing FL Ds-mEGFP (gray, *n* = 11), Ds^ΔCAD1–4^-mEGFP (dark purple, *n* = 7), or Ds^ΔCAD15–19^-mEGFP (pale purple, *n* = 10) or (J) cells expressing Ds-mApple and cells expressing FL Ft-mEGFP (gray, *n* = 10), Ft^ΔCAD1–4^-mEGFP (dark blue, *n* = 11), or Ft^ΔCAD26–30^-mEGFP (pale blue, *n* = 9). Two-phase exponential curves were fitted, and 95% confidence intervals are shown. The estimated half-life of the slow phase of recovery (90 s for FL Ds-mEGFP or 75 s for FL Ft-mEGFP) is indicated by the red line, and recovery was compared between samples at this time point (Figures S2H and S2I). (K–P) Aggregation experiments between cells expressing Ds^ΔCAD1–4^-mEGFP (K), Ds^ΔCAD15–19^-mEGFP (L), Ft^ΔCAD1–4^-mEGFP (M and O), or Ft^ΔCAD26–30^-mEGFP (N and P) and cells expressing FtCAD26–27-EGFP (K), FtCAD1–5-HA (L), DsCAD17–22-HA (M and P), or DsCAD1–4-mApple (N and O). EGFP fluorescence in green, mApple fluorescence and HA immunolabeling in magenta, and (K) immunolabeled for Ds (magenta). (Q) Scoring of percentage of cells expressing Ft^ΔCAD1–4^-mEGFP or Ft^ΔCAD26–30^-mEGFP binding to cells expressing cell surface DsCAD1–4-mApple or DsCAD17–22-HA. Error bars are SD, *n* = 3. Pre-selected pairs of samples were compared using ANOVA with Šidák’s multiple comparisons test. See also Figure S2 and Table S2.

We next investigated whether deletion/truncation of one or both of the CAD binding regions from otherwise full-length molecules affected the ability of Ds and Ft to interact in *trans* in our cell aggregation assay. Initial experiments mapped the C-terminal binding region of Ds to between CAD15 and CAD21, so we deleted CAD15–19. This deletion has incomplete overlap with the subsequently mapped minimal DsCAD17–22 binding region but nevertheless abolishes binding to Ft in combination with Ds^ΔCAD1–4^ (Figure S2A). Notably, the binding efficiency of the single deletion Ds^ΔCAD1–4^ or Ds^ΔCAD15–19^ to full-length Ft in neighboring cells was significantly reduced compared to full-length Ds (Figures 3A–3D and S2A; Table S2). Furthermore, Ds^ΔCAD1–4^ did not bind FtCAD1–5 (Figure S2C) but, as expected, still bound FtCAD26–27 (Figures 3K and S2C). Conversely, Ds^ΔCAD15–19^ did not bind FtCAD26–27 (Figure S2D) but still bound FtCAD1–5 (Figures 3L and S2D). Interestingly, although Ds^ΔCAD15–19^ bound Ft, it had reduced localization to the cell surface compared to full-length Ds or Ds^ΔCAD1–4^ (Figures 3A–3C), possibly indicating defective protein folding.

The stability of Ds-Ft interactions was previously measured using fluorescence recovery after photobleaching (FRAP),^23^ where increased mobility is a proxy for decreased binding. FRAP of Ds^ΔCAD1–4^-mEGFP or Ds^ΔCAD15–19^-mEGFP on interfaces with Ft-mApple-expressing cells revealed an increase in mobility in both cases compared to full-length Ds-mEGFP, with Ds^ΔCAD1–4^ having the stronger effect (Figures 3E and S2H; Table S2). This supports the results from the cell-binding assays (Figure 3D) showing that both binding domains of Ds contribute to the strength of Ft-Ds interactions.

Experiments were then performed using deletions of CAD1–4 and CAD26–30 of Ft, interacting with full-length Ds. Surprisingly, Ft^ΔCAD1–4^ and Ft^ΔCAD26–30^ interacted only slightly less well than full-length Ft, although again, the deletion of both regions (Ft^ΔCAD1–4ΔCAD26–30^) completely abrogated binding to Ds (Figures 3F–3I and S2B; Table S2). Furthermore, in FRAP Ft^ΔCAD1–4^-mEGFP had only a mild increase in mobility compared to full-length Ft-mEGFP, while the mobility of Ft^ΔCAD26–30^-mEGFP was unchanged (Figures 3J and S2I; Table S2). As the loss of both regions prevents the binding of Ft to full-length Ds (Figure S2B), this suggests that FtCAD1–4 and FtCAD26–30 act semi-redundantly in regulating the stability of interactions with Ds.

Surprisingly, cells expressing Ft^ΔCAD1–4^ aggregated with cells expressing either DsCAD17–22 or DsCAD1–4 (Figures 3M, 3O, and S2E). Similarly, Ft^ΔCAD26–30^ interacted with either DsCAD1–4 or DsCAD17–22 (Figures 3N, 3P, and S2F). This redundancy would explain why the deleted molecules are not defective in binding full-length Ds (Figures 3F–3J). DsCAD1–4 interacted better than DsCAD17–22 with both Ft^ΔCAD1–4^ and Ft^ΔCAD26–30^ (Figure 3Q; Table S2).

Ft^ΔCAD1–4ΔCAD26–30^ failed to interact with either DsCAD1–4 or DsCAD17–22 (Figure S2G). This suggests that there is an alternative interaction site for DsCAD1–4 within Ft^ΔCAD1–4^ overlapping the FtCAD26–30 region and an alternative interaction site for DsCAD17–22 within Ft^ΔCAD26–30^ overlapping the FtCAD1–4 region. However, we were unable to detect the binding of either DsCAD1–4 to isolated subsets of C-terminal CAD domains of Ft (Figure S1C) or DsCAD17–22 to isolated subsets of N-terminal CAD domains of Ft (Figure S1D).

Overall, our results support both N- and C-terminal CAD binding regions redundantly regulating Ft-Ds heterophilic *trans* interactions.

### The first four CAD repeats are not essential for Ft-Ds function in planar polarity

**Figure 4:**
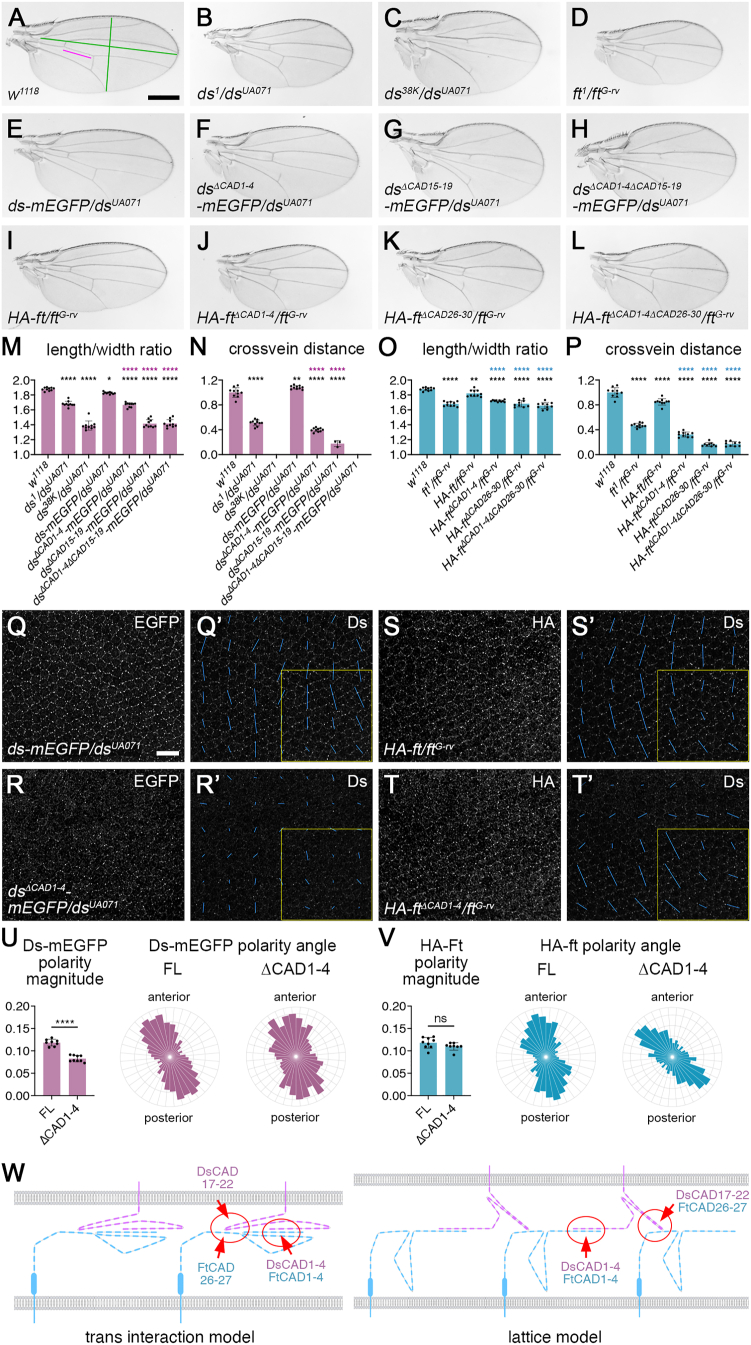
DsCAD1–4 and FtCAD1–4 are not necessary for planar polarization of Ft and Ds (A–L) Adult male wings of the indicated genotypes. Scale bar, 400 μm. (M–P) Quantitation of adult wing genotypes as shown in (A)–(L). (M and O) Length-width ratio, measured as indicated by green lines in (A). (N and P) Crossvein distance, as indicated by magenta line in (A). Error bars are SD, *n* = 10, except for (N) *ds*^*38K*^*/ds*^*UA071*^, *n* = 0; *ds*^*ΔCAD15*–*19*^*-mEGFP/ds*^*UA071*^, *n* = 3; and *ds*^*ΔCAD1*–*4ΔCAD15*–*19*^*-mEGFP/ds*^*UA071*^, *n* = 0, as most wings lacked at least one crossvein, and for (O and P) *HA-ft*^*ΔCAD26*–*30*^*/ft*^*G-rv*^, *n = 9*. Where incomplete posterior crossveins were present, crossvein distances were extrapolated. Samples were compared to *w*^*1118*^ control (black asterisks), *ds-mEGFP/ds*^*UA071*^ (purple asterisks in M and N), or *HA-ft/ft*^*G-rv*^ (blue asterisks in O and P) using ANOVA with Dunnett’s multiple comparisons test (^∗^*p* < 0.05, ^∗∗^*p* < 0.01, and ^∗∗∗∗^*p* ≤ 0.0001). (Q–T) 28 h after puparium formation (APF) pupal wings from *ds-mEGFP/ds*^*UA071*^ (Q), *ds*^*ΔCAD1*–*4*^*-mEGFP/ds*^*UA071*^ (R), *HA-ft/ft*^*G-rv*^ (S), or *HA-ft*^*ΔCAD1*–*4*^*/ft*^*G-rv*^ (T) flies. Images show EGFP fluorescence (Q and R), HA immunolabeling (S and T), or Ds immunolabeling and nematics for neighbor vector polarity magnitude (Q′–T′). Scale bar, 10 μm. (U and V) Cell-by-cell polarity measurements based on Ds immunolabeling in region near wing margin marked in yellow in (Q′)–(T′) for *ds-mEGFP* variants (U) or *HA-ft* variants (V). Error bars are SD, *n* = 8 (Ds-FL), *n* = 9 (Ds^ΔCAD1–4^), *n* = 9 (Ft-FL), and *n* = 8 (Ft^ΔCAD1–4^). Polarity magnitudes were compared using an unpaired t test (^∗∗∗∗^*p* ≤ 0.0001). Rose plots show the distribution of polarity angles pooled for all wings. Note that the polarity angle of the *HA-ft*^*ΔCAD1–4*^ wings shifts slightly toward proximal-distal, but this may be due to the different wing shape or size. (W) Model of Ft-Ds heterophilic binding based on the data in this manuscript and the structure of mammalian Dchs1 and Fat4 revealed by cryo-EM imaging and atomic modeling.^17^ Left: *trans* interactions between N-terminal and C-terminal CAD binding sites in the same molecules. Right: lattice model, whereby *trans* interactions between N-terminal CAD binding sites and C-terminal CAD binding sites in different molecules promote *cis* clustering. See also Figures S3 and S4 and Table S2.

We then tested the effects of deleting the Ft and Ds binding domains in flies. Rescue transgenes consisting of full-length or deleted forms of Ft and Ds were inserted into the genomic loci^30^ (see STAR Methods), tagged with either HA or mEGFP. *ds-mEGFP* rescued the defects in wing shape associated with *ds* mutants (Figures 4A–4C, 4E, 4M, 4N, and S3E), while *HA-ft* rescued the viability and wing shape of *ft* mutants, but the wings were slightly undergrown (Figures 4A, 4D, 4I, 4O, 4P, and S3F).

*ds*^*ΔCAD1–4*^*-mEGFP* flies had rounder wings than normal, with a reduced distance between the crossveins, phenotypes typical of weak *ds* mutants (Figures 4B, 4F, 4M, 4N, and S3E; Table S2). In contrast, *ds*^*ΔCAD15–19*^*-mEGFP* and *ds*^*ΔCAD1–4ΔCAD15–19*^*-mEGFP* flies had a much stronger defect in wing shape and crossvein distance, as well as trichome orientation defects typical of strong *ds* mutants (Figures 4C, 4G, 4H, 4M, 4N, S3A, S3B, and S3E; Table S2). *HA-ft*^*ΔCAD1–4*^, *HA-ft*^*ΔCAD26–30*^, or *HA-ft*^*ΔCAD1–4ΔCAD26–30*^ flies were rescued to viability, consistent with the rescue of Hippo-Warts-mediated overgrowth, and again had rounder wings with reduced crossvein distance (Figures 4J–4L, 4O, 4P, and S3F; Table S2). *HA-ft*^*ΔCAD26–30*^ and *HA-ft*^*ΔCAD1–4ΔCAD26–30*^ flies also had weak trichome orientation defects in the proximal wing (Figures S3C and S3D), similar to previously reported deletions of the extracellular domain of Ft.^29^ A small number of *HA-ft*^*ΔCAD26–30*^ or *HA-ft*^*ΔCAD1–4ΔCAD26–30*^ animals had a more extended wing shape (Figures S3G–S3I). These flies appeared to belong to a distinct phenotypic class (Figure S3I) and were excluded from our main analysis.

For both Ds and Ft, deletion of CAD1–4 gave a weaker phenotype than deletion of the C-terminal CAD binding sites (Figures 4M–4P; Table S2), which was surprising as the experiments in cultured cells suggested that deletion of CAD1–4 was more deleterious. Moreover, simultaneous deletion of CAD1–4 from both Ft and Ds gave similar phenotypes to deleting either by itself (Figures S3J–S3M; Table S2).

We then examined subcellular localization of the mutated molecules in pupal wings. Strikingly, deletion of DsCAD1–4, FtCAD1–4, or both did not abolish the binding between Ds and Ft. Both molecules still localized with a punctate distribution to cell junctions, suggestive of heterophilic binding interactions, and polarity was perpendicular to the wing margin, similar to full-length molecules (Figures 4Q–4V and S3N-P). Polarity strength was reduced for Ds^ΔCAD1–4^ but not for Ft^ΔCAD1–4^ (Figures 4U and 4V; Table S2), in keeping with the lesser effect of deleting FtCAD1–4 in cell culture (Figures 3I and 3J). This indicates that DsCAD1–4 and FtCAD1–4 are not essential for binding or planar polarity.

Ds^ΔCAD1–4^ and Ft^ΔCAD1–4^ were excluded from clone boundaries when they were adjacent to endogenous Ds or Ft (compare Figures S4A, S4B, S4D, and S4E). This indicates that they are outcompeted by wild-type Ds and Ft and suggests that both molecules have a reduced binding ability compared to full-length molecules. Conversely, both were recruited to clone boundaries when they were adjacent to tissue lacking *ds* or *ft*, respectively (Figures S4G, S4H, S4J, and S4K), consistent with them retaining some heterophilic *trans*-binding activity.

Ds^ΔCAD15–19^ was excluded from apicolateral cell junctions, and there was also no strong enrichment of Ft^ΔCAD26–30^ at cell junctions (Figures S4C, S4F, and S4I), suggesting no significant heterophilic binding activity. This is consistent with the strong phenotypes observed in adult wings but is surprising because the cell culture data suggest that binding activity is retained. However, Ft^ΔCAD26–30^ was weakly enriched at clone boundaries adjacent to tissue lacking *ft* (Figure S4L), suggesting that it retains some ability to interact with Ds in neighboring cells when not competing with endogenous Ft.

In summary, our results indicate that planar polarity is partially rescued in *ds*^*ΔCAD1–4*^ and *ft*^*ΔCAD1–4*^ flies. This is consistent with a model in which CAD1–4 from both molecules contribute to their heterophilic binding, but additional heterophilic interactions contribute to Ft-Ds binding at cell-cell interfaces and planar polarity function.

## Discussion

In this work, we demonstrate that two distinct CAD regions contribute to the heterophilic binding between Ft and Ds. Previous studies have identified N-terminal binding sites, whereby CAD1–4 of Ds bind CAD1–4 of Ft.^17^^,^^21^^,^^22^^,^^24^ Using a cell aggregation assay, we now show that CAD17–22 of Ds interact with CAD26–27 of Ft. Importantly, we demonstrate that the two binding sites are conserved in hDchs1 and hFat4. We provide *in vivo* evidence that both binding sites are important for heterophilic interactions and contribute to planar polarity activity in flies.

We propose a model whereby the C-terminal CAD binding site acts to increase the strength of Ft-Ds binding. Interactions between the N-terminal CAD sites could initiate the binding, and this could be stabilized by subsequent interactions between the C-terminal CAD sites, or vice versa. This could assist with the packing of the molecules into the extracellular space. Another possibility is that the presence of two binding sites could contribute to the clustering of Ft and Ds: binding between FtCAD1–4 and DsCAD1–4 could be accompanied by DsCAD17–22 interacting with CAD26–30 of a different Ft molecule (Figure 4W). This would promote a lattice-type arrangement of Ft and Ds molecules, which could contribute to the concentration into junctional puncta seen *in vivo.*^30^

What is the function of the remaining CAD repeats? They may have a structural role: the *trans*-interacting repeats will most likely need to be precisely positioned relative to each other and the plasma membrane, which would depend on the remaining repeats maintaining their rigid structures and having either rigid or flexible linkers in the correct positions in the chain.^17^ Secondly, some CAD repeats could be involved in *cis* interactions, which promote the clustering of molecules. Clustering may also be enhanced by dimerization of the intracellular domains, previously demonstrated for Ft^31^ and which we also detect for both Ft and Ds using a *cis*-recruitment cell aggregation assay (Figures S4M–S4S).

We also demonstrate that neither DsCAD1–4 nor FtCAD1–4 are necessary in flies, as *ds*^*ΔCAD1–4*^ and *ft*^*ΔCAD1–4*^ flies still exhibit Ft-Ds *trans* binding and planar polarity (albeit weaker than normal for *ds*^*ΔCAD1–4*^). Conversely, deleting the C-terminal CAD binding sites had strong effects on Ft-Ds binding in flies. *ds*^*ΔCAD15–19*^ behaved like a null allele in flies, and as the protein was also poorly localized to the cell surface in cell culture, it may be mis-folded. Ft^ΔCAD26–30^, on the other hand, retained very weak binding to Ds in pupal wings and rescued lethality and overgrowth, suggesting functional interactions with downstream Hippo-Warts signaling. The strong effect of Ft^ΔCAD26–30^ on Ft-Ds binding in flies was surprising, as there was no significant effect in cell culture. Similarly, the mild phenotype of *ds*^*ΔCAD1–4*^ in flies contrasts with the greatly reduced binding of Ds^ΔCAD1–4^ to Ft in tissue culture. It may be that in flies, the loss of CAD1–4 has less of an effect, as the existing cell junctions bring the C-terminal CAD sites closer together. Alternatively, heterophilic binding could be affected by the more complex environment of intercellular junctions *in vivo*.

Using domain deletion and swapping experiments, we show in cell culture that DsCAD1–4 bind to FtCAD1–4 in an anti-parallel arrangement, in agreement with a recent crystal structure of the mammalian homologs.^24^ We also demonstrate that all four CADs contribute to the binding efficiency. DsCAD1–2 binding to FtCAD3–4 appears to be more important than DsCAD3–4 binding to FtCAD1–2. Interestingly, a more important role for DsCAD1–2 binding to FtCAD3–4 is not predicted from the crystal structure, which shows minimal contacts between CAD2 of Dchs1 and CAD3 of Fat4.^24^

Future studies will be needed to understand the binding of DsCAD17–22 to FtCAD26–27. It is unclear how six CAD domains of Ds can interact with just two CAD domains of Ft. Interestingly, cryoelectron microscopy (cryo-EM) images and atomic modeling of vertebrate Dchs1 predict a strong kink between CAD19 and CAD20.^17^ This is predicted to cause the molecule to fold back on itself, and the resulting 3D structure may generate a novel motif for interacting with FtCAD26–27.

### Limitations of the study

Our study used heterologous TM constructs containing subsets of Ft or Ds CAD repeats to map *trans* interactions. We cannot exclude the possibility that there are additional *trans* interaction sites that we have not identified, as some groups of repeats may not be in a suitable context for correct folding and surface presentation. The apparent semi-redundancy of DsCAD1–4 and DsCAD17–22 binding to full-length Ft may also be an effect of overexpression in this artificial assay. We also could not conclusively prove a role for the C-terminal Ds binding site, as alleles in which this site was deleted behaved as null mutations.

## Resource availability

### Lead contact

Further information and requests for resources and reagents should be directed to and will be fulfilled by the lead contact, Helen Strutt (h.strutt@sheffield.ac.uk).

### Materials availability

Fly strains and plasmids reported in this paper will be shared by the lead contact upon request.

### Data and code availability

All data reported in this paper will be shared by the lead contact upon request. This paper does not report original code. Any additional information required to reanalyze the data reported in this paper is available from the lead contact upon request.

## Acknowledgments

We thank the Bloomington Drosophila Stock Center for fly stocks, Genetivision for the generation of transgenics, and David Sprinzak for hFat4 and hDchs1 cDNAs. Amy Brittle is thanked for making the initial tissue culture vectors, constructs, and flies; Varun Chaudhary for making the Actin-FLP construct; and Larra Trinidad for help with the rose plots. We thank Rob Hunton and Larra Trinidad for comments on the manuscript and the fly room staff for excellent technical support. Imaging was performed in the Wolfson Light Microscopy Facility. The work was funded by a 10.13039/501100000268BBSRC grant to D.S. and H.S. (BB/S001395/1) and a Wellcome Trust Senior Fellowship award to D.S. (210630/Z/18/Z).

## Author contributions

Conceptualization, D.S.; methodology, D.S. and H.S.; formal analysis, D.M. and H.S.; investigation, A.C.K.M., D.M., E.M., and H.S.; writing – original draft, H.S.; writing – review & editing, D.S. and H.S.; visualization, D.M. and H.S.; supervision, D.S. and H.S.; funding acquisition, D.S. and H.S.

## Declaration of interests

The authors declare no competing interests.

## STAR★Methods

### Key resources table

**Table:** 

REAGENT or RESOURCE	SOURCE	IDENTIFIER
**Antibodies**		

Rat monoclonal anti-HA 3F10	Roche	cat#1867431; RRID: AB_390918
Mouse monoclonal anti-HA 16B12	BioLegend	cat#901502; RRID: AB_2565007
Affinity-purified rabbit anti-Ds	Strutt and Strutt^14^	N/A
Affinity-purified rabbit anti-Ft	Brittle et al.^12^	N/A

**Chemicals, peptides, and recombinant proteins**		

Schneider’s Drosophila medium	Gibco	cat#21720024
Heat-inactivated fetal bovine serum	Gibco	cat#10082-147
Penicillin-Streptomycin	Sigma-Aldrich	cat#P4333
Effectene transfection reagent	Qiagen	cat#301425
Paraformaldehyde	Agar Scientific	cat#AGR1026
Normal goat serum	Jackson ImmunoResearch	cat#005-000-121; RRID:AB_2336990
Prolong Diamond	Thermo Fisher Scientific	cat#P36965

**Experimental models: Cell lines**		

*D. melanogaster*: Cell line S2	DGRC: 6	FLYB:FBtc0000006

**Experimental models: Organisms/strains**		

*D. melanogaster*: *ds[1]*	Clark et al.^4^	BDSC:3446; FLYB:FBal0003119
*D. melanogaster*: *ds[UA071]*	Adler et al.^33^	BDSC:41784; FLYB:FBal0089339
*D. melanogaster*: *ds[38K]*	Clark et al.^4^	BDSC:288; FLYB:FBal0028156
*D. melanogaster*: *ft[1]*	Mohr^32^	BDSC:304; FLYB:FBal0004787
*D. melanogaster*: *ft[G-rv]*	Mahoney et al.^5^	BDSC: 1894; FLYB:FBal0004805
*D. melanogaster*: *ft-EGFP*	Hale et al.^23^	PMID:25707557
*D. melanogaster*: *ds-mApple*	Brittle et al.^30^	PMID:36170824
*D. melanogaster*: *ds-mEGFP*	This paper	N/A
*D. melanogaster*: *ds*^*ΔCAD1-4*^*-mEGFP*	This paper	N/A
*D. melanogaster*: *ds*^*ΔCAD5-19*^*-mEGFP*	This paper	N/A
*D. melanogaster*: *ds*^*ΔCAD1-4ΔCAD*^^*1*^^*5-19*^*-mEGFP*	This paper	N/A
*D. melanogaster*: *HA-ft*	This paper	N/A
*D. melanogaster*: *HA-ft*^*ΔCAD1-4*^	This paper	N/A
*D. melanogaster*: *HA-ft*^*ΔCAD26-30*^	This paper	N/A
*D. melanogaster*: *HA-ft*^*ΔCAD1-4ΔCAD26-30*^	This paper	N/A
*D. melanogaster*: *P[w+, arm-lacZ] FRT40*	Bloomington Drosophila Stock Center	BDSC:7371; RRID:BDSC_7371
*D. melanogaster*: *Ubx-FLP* on X	Bloomington Drosophila Stock Center	BDSC:42718; FLYB:FBti0150334

**Recombinant DNA**		

pMK33β-CD2[Sig]-CAD-CD2[TM+Intra]-EGFP	This paper	N/A
pMK33β-CD2[Sig]-CAD-CD2[TM+Intra]-3xHA	This paper	N/A
pMK33β-CD2[Sig]-CAD-CD2[TM+Intra]-mApple	This paper	N/A
pMK33β-CD2[Sig+TM]-ds[ICD]-EGFP	This paper	N/A
pMK33β-CD2[Sig+TM]-ft[ICD]-EGFP	This paper	N/A
pAttB-ActP-FRT-polyA-FRT-dsCAD1-27	This paper	N/A
pAttB-ActP-FRT-polyA-FRT-ftCAD1-34	This paper	N/A
pActin-FLP	This paper	N/A
pGE-MT-ds-mEGFP	This paper	N/A
pGE-MT-ds-mApple	This paper	N/A
pGE-MT-ds^ΔCAD1^-mEGFP	This paper	N/A
pGE-MT-ds^ΔCAD1-2^-mEGFP	This paper	N/A
pGE-MT-ds^ΔCAD1-4^-mEGFP	This paper	N/A
pGE-MT-ds^ΔCAD15-19^-mEGFP	This paper	N/A
pGE-MT-ds^ΔCAD1-4ΔCAD15-19^-mEGFP	This paper	N/A
pGE-MT-ft-mEGFP	This paper	N/A
pGE-MT-ft-mApple	This paper	N/A
pGE-MT-ft^ΔCAD1^-mEGFP	This paper	N/A
pGE-MT-ft^ΔCAD1-2^-mEGFP	This paper	N/A
pGE-MT-ft^ΔCAD1-4^-mEGFP	This paper	N/A
pGE-MT-ft^ΔCAD26-30^-mEGFP	This paper	N/A
pGE-MT-ft^ΔCAD1-4ΔCAD26-30^-mEGFP	This paper	N/A

**Software and algorithms**		

ImageJ version 2.0.0-rc-69/1.52p	https://fiji.sc	N/A
GraphPad Prism version 9	www.graphpad.com	N/A
Tissue Analyzer	Aigouy et al.^9^	PMID:20813263
QuantifyPolarity version 9	Tan et al.^40^	PMID:34351416

### Experimental model and study participant details

#### Flies

*Drosophila melanogaster* lines were grown on standard cornmeal/agar/molasses media at 25°C. Male flies were selected for analysis of adult wing size and shape, as male and females have different body sizes. There are no known differences in the physical and molecular mechanisms of planar polarity in male and female flies, thus flies were not distinguished based on sex for pupal wing experiments. Fly strains are described in FlyBase. *ds*^*1*^ and *ft*^*1*^ are hypomorphic alleles,^4^^,^^32^ while *ds*^*UA071*^, *ds*^*38K*^ and *ft*^*G-rv*^ are strong alleles that produce no detectable protein.^4^^,^^5^^,^^33^ *ft-EGFP*^23^ and *ds-mApple*^30^ were previously described.

#### Cell culture

S2 cells, of unknown sex, were cultured in Schneider’s *Drosophila* medium, supplemented with 10% heat inactivated fetal bovine serum and 1% Penicillin-Streptomycin at 26°C.

### Method details

#### Molecular biology

Subsets of CAD repeats of Ds and Ft were isolated by PCR and cloned into the *pMK33ß* vector, downstream of a CD2 signal sequence and upstream of a region spanning the rat CD2 transmembrane domain (amino acids 189–254), with C-terminal tags of EGFP, mApple or 3x-HA. DsCAD1-27 and FtCAD1-34 were cloned in *pAttB-ActP-FRT-polyA-FRT*.^34^ Chimaeras consisting of alternating Ds and Ft CAD repeats were generated using overlap PCR and cloned into the same *pMK33ß* vector. ICDs of Ft and Ds were tagged with EGFP and cloned downstream of the CD2 signal sequence and transmembrane domain (amino acids 189–225). CAD repeats were identified using Prosite, and specific amino acids included in each construct are in Table S1, based on the NP_523446 Ds, NP_477497 Ft, Q96JQ0 hDchs1 or Q6V0I7 hFat4 protein accession sequences. *pKS-Actin-FLP* was generated by cloning the yeast *FLP* gene between a *Drosophila Actin5C* promoter and an SV40 *polyA* sequence, in *pBluescript KS+*.

For *ft-mEGFP, ft-mApple* and *HA-ft*, a *ft* cDNA with 56 bp of 5′ UTR and the complete *ft* 3′ UTR was cloned into a version of the vector *pGE-attB-GMR*^35^ that was modified to permit recombineering. *ft* was then tagged with mEGFP or mApple at the C terminus or HA at the N terminus after the signal sequence. *ds-mEGFP* and *ds-mApple* were made by inserting a *ds* cDNA fused to C-terminal mEGFP or mApple tags, with 50 bp of 5′ UTR and the complete *ds* 3′ UTR. For tissue culture experiments, the 5′ UTRs were replaced by the metallothionein promoter. CAD deletions were generated using standard recombineering methods.^36^

#### Generation of transgenic flies

Transgenic fly lines were generated for this study by injection of constructs by Genetivision. An *attP* site was inserted into the *ft* locus, deleting from 56 bp upstream of the ATG to 5.5 kb downstream, and removing the entire coding sequence of the first coding exon, using the targeting vector *pTV[Cherry]*.^37^ Rescue constructs expressing *HA-ft* variants and *ds-mEGFP* variants were then inserted into the *ft attP* site, or a previously generated *ds attP* site.^30^ Transgenes were recombined onto *FRT40*,^38^ and clones were made using *Ubx-FLP*.^39^

#### Antibodies

Primary antibodies used for immunolabelling were rat anti-HA 3F10 (Roche cat#1867431), mouse anti-HA 16B12 (BioLegend cat#901513), affinity-purified rabbit anti-Ds^14^ and affinity-purified rabbit anti-Ft.^12^

#### Transfection of cells

Cells were transfected using Effectene transfection reagent (Qiagen), according to the manufacturer’s instructions. Expression was induced by addition of 350 μM CuSO_4_ to the culture medium for 20–24 h. For DsCAD1-27 and FtCAD1-34, *pKS-Actin-FLP* was co-transfected to excise the *FRT-STOP-FRT* cassette.

#### Cell aggregation experiments

Transfected cells in 350 μM CuSO_4_ were washed with culture medium and diluted to 8 x 10^5^/mL in media containing 350 μM CuSO_4_. 250 μL of each cell type (500 μL total) was placed into wells of a non-treated 24 well plate. Cells were allowed to aggregate by swirling at 110 rpm for 90–120 min at 26°C. Cells were then transferred using a 1000 μL pipette with a cut-off tip, onto 13 mm coverslips in a fresh 24 well plate, or onto the center of CellView cell culture dishes (Greiner) for FRAP. The original wells were washed with 100 μL media containing 350 μM CuSO_4_, and this was added to the coverslips or cell culture dishes. Cells were allowed to settle for 2 h before further processing. For FRAP a further 1 mL of media containing 350 μM CuSO_4_ was added to the CellView dishes immediately before imaging, so that the media covers the entire surface of the dish.

#### Immunolabelling of cells

Cells on coverslips were washed briefly in PBS, then fixed for 20 min in 4% paraformaldehyde in PBS. They were blocked for 1 h in PBS containing 0.2% Triton X-100 (PTX) and 10% normal goat serum. Primary antibodies were incubated overnight at 4°C, and secondary antibodies for 2–4 h at RT, in PTX with 10% normal goat serum, and all washes were in PTX. After immunolabelling, wings were post-fixed in 4% paraformaldehyde in PBS for 10 min, and mounted in ProLong Diamond.

#### FRAP

For FRAP, cells in Schneider’s medium were plated into CellView dishes and imaged on a Nikon A1 GaAsP confocal microscope. Images were 512 x 512 pixels, with a pixel size of 80 nm, and a pinhole of 1.2 AU. Elliptical ROIs of 3–4 μm^2^ were selected, on cell boundaries where cells expressing EGFP-tagged proteins formed interfaces with cells expressing mApple-tagged proteins. Three pre-bleach images were taken at 2 frames/sec, and ROIs were then bleached using a single pass of a 488 nm Argon laser at 8%, which resulted in 60–75% bleaching. Immediately following bleaching, 5 images were taken at 5 s intervals, followed by 10 images at 10 s intervals and 26 images at 15 s intervals.

#### Adult wings

Adult wings were dehydrated in isopropanol, mounted in Gary’s Magic Mountant (50% methyl salicylate, 50% Canada balsam) and left to clear overnight at 60°C.

#### Wing disc and pupal wing dissection and immunolabelling

Wing discs from wandering third-instar larvae were dissected in PBS, and fixed in 4% paraformaldehyde for 20 min at room temperature. Pupal wings were dissected at 28 h after puparium formation (APF) at 25°C. Briefly, pupae were removed from their pupal case and fixed for 35–40 min in 4% paraformaldehyde in PBS. Wings were then dissected and the outer cuticle removed. Tissues were blocked for 1 h in PTX and 10% normal goat serum. Primary and secondary antibodies were incubated overnight at 4°C in PTX with 10% normal goat serum, and all washes were in PTX. After immunolabelling, wings were post-fixed in 4% paraformaldehyde in PBS for 30 min. Pupal wings were mounted in 25 μL PBS containing 10% glycerol and 2.5% DABCO, pH7.5, and wings discs were mounted in 20 μL Mowiol.

### Quantification and statistical analysis

#### Quantitation of cell aggregation experiments

Aggregation experiments were scored by manual counting using an epifluorescence microscope. Cells transfected with plasmids tagged with EGFP were mixed with cells transfected with plasmids tagged with mApple or HA, where HA was immunolabelled with Alexa Fluor 568. 100–200 cells transfected with one plasmid were examined and the percentage of cells binding to the other cell type was counted. Samples were compared using ANOVA with Dunnett’s multiple comparisons test, or pre-selected pairs of samples were compared using ANOVA with Šidák’s multiple comparisons test.

#### FRAP analysis

ImageJ was used to manually reselect and measure bleached ROIs of 1.5–2 μm^2^ in each image for each time point. The laser off background was subtracted, and the values were normalised against the average of the prebleach values. We found no evidence for acquisition bleaching during the timecourse of FRAP experiments in cell culture, so no correction was made. Data were then plotted on an xy graph using Prism (v9 Graphpad), and one-phase exponential curves were fitted to check for goodness of fit. Curves were excluded if the ROI recovery curve failed the "replicates test for lack of fit" in Prism. Data from multiple ROIs from different interfaces were then combined and two-phase exponential association curves were fitted.

Fluorescence recovery was still ongoing at the end of the experiment, and it was not feasible to carry out FRAP for long enough for recovery to reach a plateau. To quantitatively compare between samples, the amount of recovery was measured at a fixed time point, that was equal to the estimated half-life of the slow recovery phase of the wild-type control (90 s for Ds-mEGFP, or 75 s for Ft-mEGFP). This allowed us to distinguish between genotypes with slow recovery and those with fast recovery. Recoveries were compared using ANOVA with Dunnett’s multiple comparison test.

#### Measurement of adult wings

Measurements of photomicrographs of adult wings were made in ImageJ. Samples were compared using ANOVA with Dunnett’s multiple comparisons test.

#### Quantitation of polarity

Membrane masks were generated in Tissue Analyzer,^9^ and polarity measurements made using QuantifyPolarity.^40^ Cell-by-cell polarity magnitudes and angles were determined using the Principal Component Analysis (PCA) method, and nematics are average vector polarity (coarse-grain polarity), based on 3x3 groups of cells. Samples were compared using unpaired t-tests.

## Supplemental information

Document S1. Figures S1–S4 and Tables S1 and S2

Document S2. Article plus supplemental information
