# Supplementary material for: Fat-Dachsous planar polarity function requires two distinct heterophilic cadherin-cadherin binding interactions
Source: Cell Rep. 2024 Sep 19;43(10):114722. doi: 10.1016/j.celrep.2024.114722 (PMC11497213; doi:10.1016/j.celrep.2024.114722)
Supplement: Document S1. Figures S1–S4 and Tables S1 and S2 [file mmc1.pdf]

Cell Reports, Volume *43*

Supplemental information

Fat-Dachsous planar polarity function requires two distinct heterophilic cadherin-cadherin binding interactions

Helen Strutt, Dipak Meshram, Elizabeth Manning, Amritha Chemmenchery Kokkam Madathil, and David Strutt

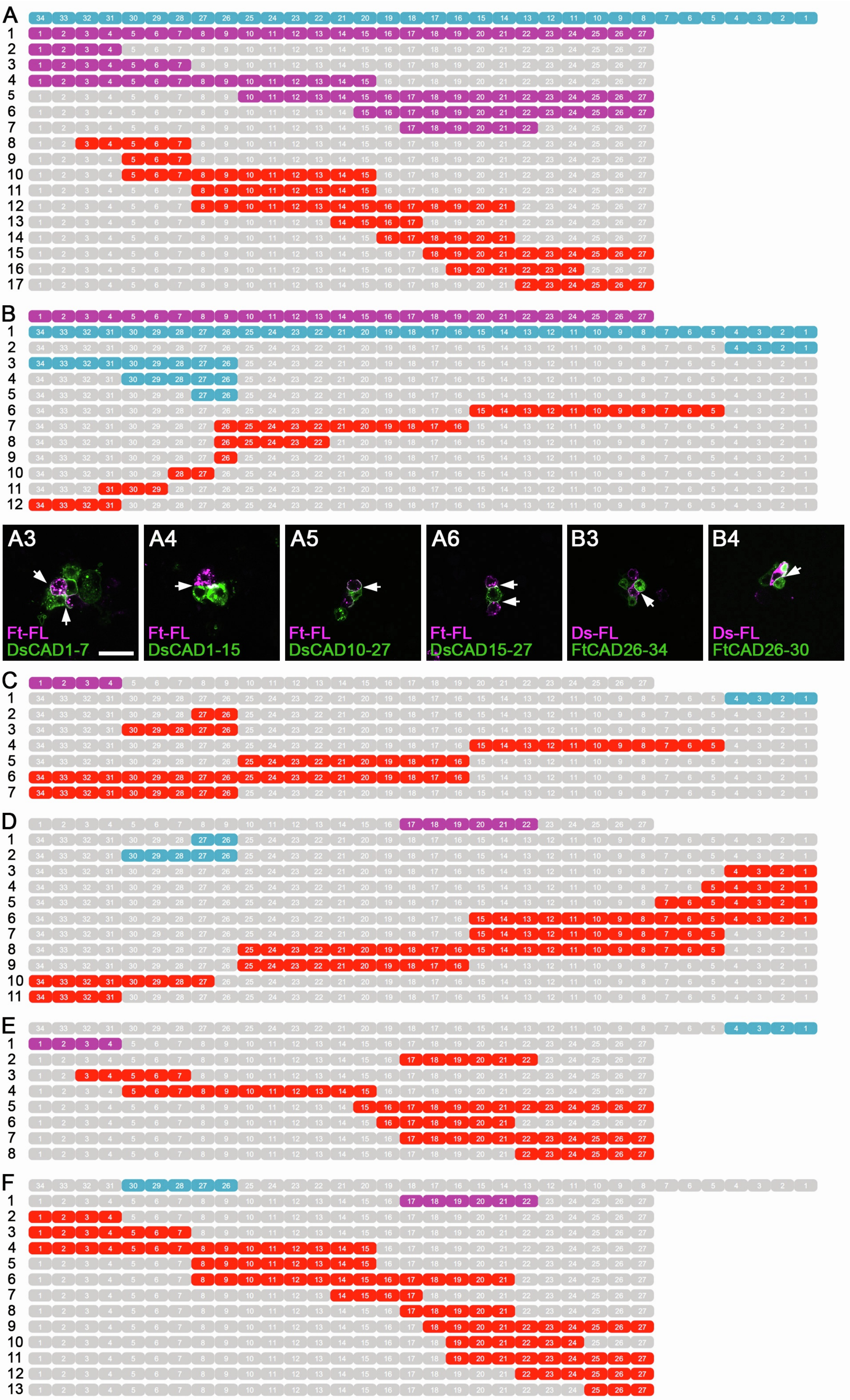
A
B
A3
Ft-FL
DsCAD1-7
A4
Ft-FL
DsCAD1-15
A5
Ft-FL
DsCAD10-27
A6
Ft-FL
DsCAD15-27
B3
Ds-FL
FtCAD26-34
B4
Ds-FL
FtCAD26-30
C
D
E
F

Figure S1. Dissecting heterophilic binding of Ds and Ft CAD repeats. Related to Figure 1.

(A) Diagrams illustrating binding of cells expressing full-length Ft-mApple (cyan) to cells expressing cell surface Ds CAD repeats as indicated. Purple indicates sets of Ds CAD repeats that bind to full-length Ft, and red indicates sets of CAD repeats that do not bind.

(B) Diagrams illustrating binding of cells expressing full-length Ds-mApple (purple) to cells expressing cell surface Ft CAD repeats as indicated. Cyan indicates sets of Ft CAD repeats that bind to full-length Ds, and red indicates sets of CAD repeats that do not bind.

(A3-A6) Aggregation experiments between cells expressing full-length Ft-mApple and cells expressing EGFP-tagged cell surface CAD repeats DsCAD1-7 (A3), DsCAD1-15 (A4), DsCAD10-27 (A5) or DsCAD15-27 (A6). Arrows point to interfaces between Ds- and Ft-expressing cells. Scale bar 20 μm.

(B3, B4) Aggregation experiments between cells expressing full-length Ds-mApple and cells expressing EGFP-tagged cell surface CAD repeats FtCAD26-34 (B3) or FtCAD26-30 (B4). Arrows point to interfaces between Ds- and Ft-expressing cells.

(C-F) Diagrams illustrating binding of cells expressing cell surface Ds CAD repeats (purple) and cells expressing cell surface Ft CAD repeats (cyan) as indicated. Red indicates sets of CAD repeats that do not bind.

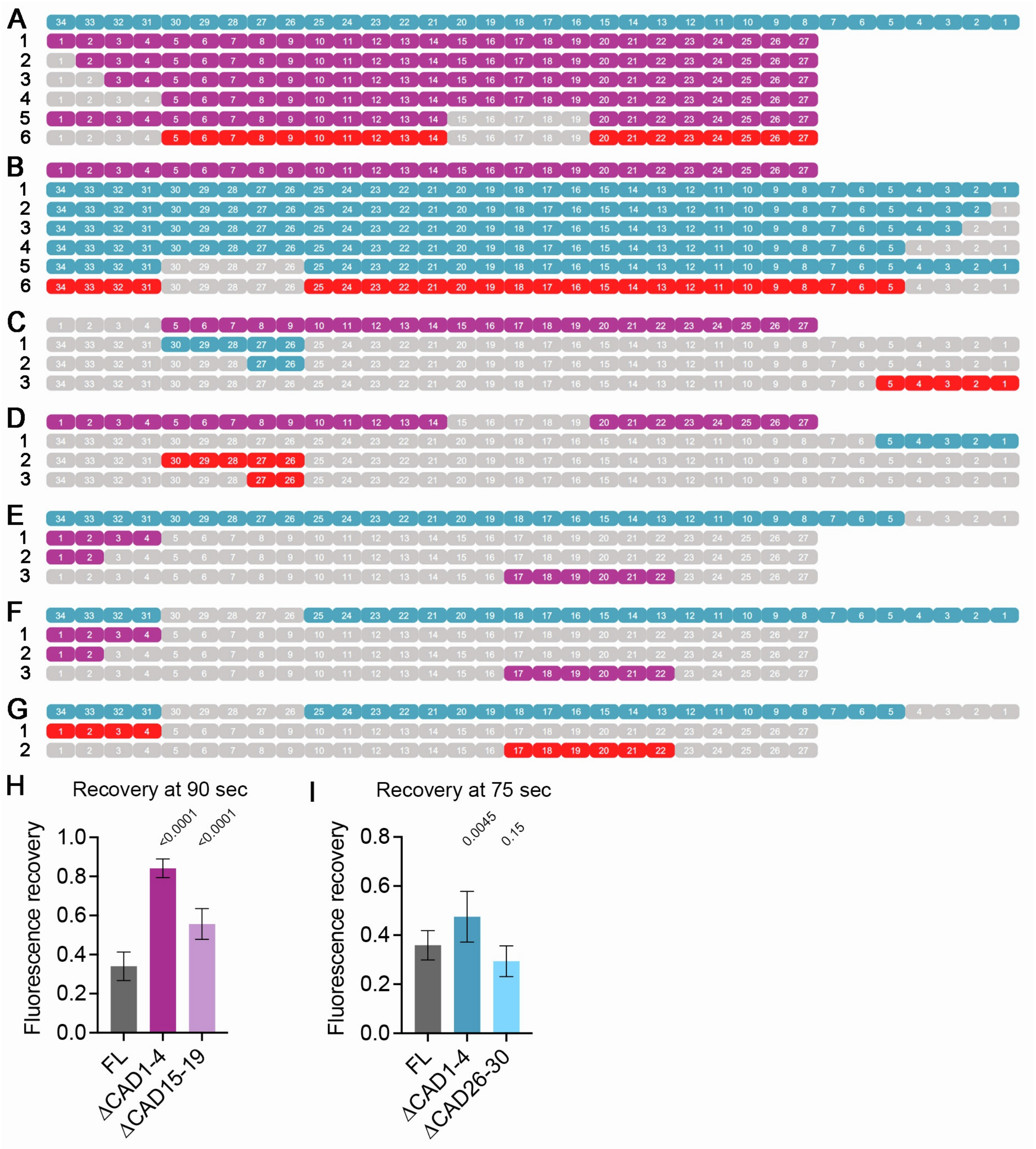

Figure S2. Dissecting the contributions of N-terminal and C-terminal CAD binding sites to the stability of Ft-Ds interactions. Related to Figure 3.

(A) Diagrams illustrating binding of cells expressing full-length Ft-mApple (cyan) to cells expressing Ds-mEGFP in which CAD repeats have been deleted, as indicated. Purple indicates Ds molecules that bind to full-length Ft, and red indicates Ds molecules that do not bind.

(B) Diagrams illustrating binding of cells expressing full-length Ds-mApple (purple) to cells expressing Ft-mEGFP in which CAD repeats have been deleted, as indicated. Cyan

indicates Ft molecules that bind to full-length Ds, and red indicates Ft molecules that do not bind.

(C, D) Diagrams illustrating binding of cells expressing $Ds^{\Delta CAD1\text{-}4}$-mEGFP (C, purple) or $Ds^{\Delta CAD15\text{-}19}$-mEGFP (D, purple) to cells expressing cell surface Ft CAD repeats as indicated. Cyan indicates sets of Ft CAD repeats that bind to $Ds^{\Delta CAD}$-mEGFP, and red indicates sets of CAD repeats that do not bind.

(E-G) Diagrams illustrating binding of cells expressing $Ft^{\Delta CAD1\text{-}4}$-mEGFP (E, cyan), $Ft^{\Delta CAD26\text{-}30}$-mEGFP (F, cyan) or $Ft^{\Delta CAD1\text{-}4\Delta CAD26\text{-}30}$-mEGFP (G, cyan) to cells expressing cell surface Ds CAD repeats as indicated. Purple indicates sets of Ds CAD repeats that bind to $Ft^{\Delta CAD}$-mEGFP, and red indicates sets of CAD repeats that do not bind.

(H,I) Fluorescence recovery of Ds-mEGFP variants on interfaces next to cells expressing Ft-mApple at 90 sec after photobleaching (H), or Ft-mEGFP variants on interfaces next to cells expressing Ds-mApple at 75 sec after photobleaching (I), which is the estimated slow half-life of full-length Ds-mEGFP and FL Ft-mEGFP, respectively (see Fig.3E,J). Absolute rates of recovery or plateaux cannot be determined as the recovery is not complete at the end of the experiment. Recovery at a defined timepoint is therefore used to compare the deleted molecules to the full-length molecules, using ANOVA with Dunnett's multiple comparisons test. Error bars are SD. (H) Ds-FL-mEGFP (n=11), $Ds^{\Delta CAD1\text{-}4}$-mEGFP (n=7) or $Ds^{\Delta CAD15\text{-}19}$-mEGFP (n=10). (I) Ft-FL-mEGFP (n=10), $Ft^{\Delta CAD1\text{-}4}$-mEGFP (n=11) or $Ft^{\Delta CAD26\text{-}30}$-mEGFP (n=9).

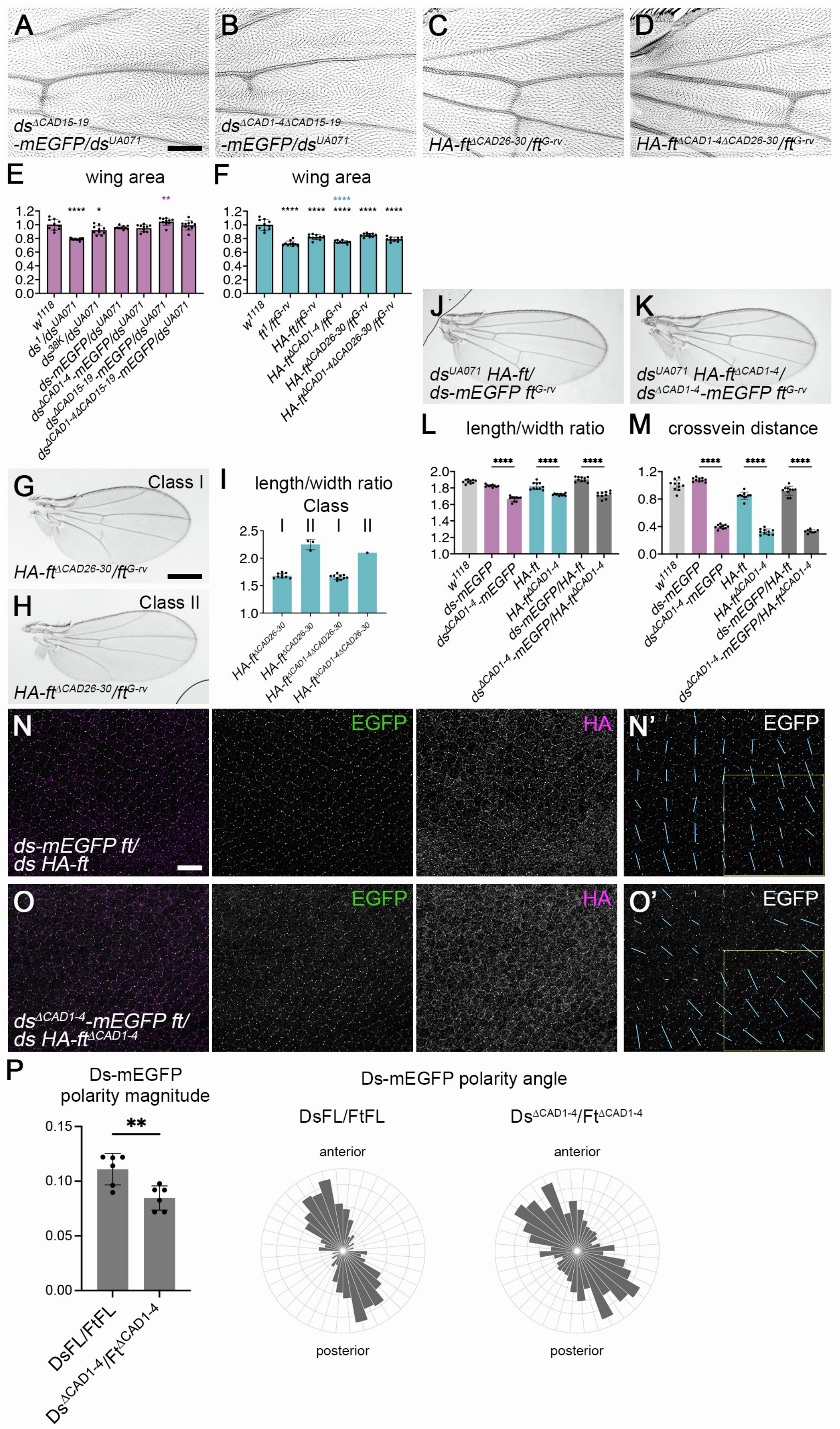
A
dsΔCAD15-19-mEGFP/dsUA071
B
dsΔCAD1-4ΔCAD15-19-mEGFP/dsUA071
C
HA-ftΔCAD26-30/ftG-rv
D
HA-ftΔCAD1-4ΔCAD26-30/ftG-rv
E
wing area
F
wing area
G
Class I
HA-ftΔCAD26-30/ftG-rv
H
Class II
HA-ftΔCAD26-30/ftG-rv
I
length/width ratio
Class
J
dsUA071 HA-ft/
ds-mEGFP ftG-rv
K
dsUA071 HA-ftΔCAD1-4/
dsΔCAD1-4-mEGFP ftG-rv
L
length/width ratio
M
crossvein distance
N
ds-mEGFP ft/
ds HA-ft
EGFP
HA
N'
O
dsΔCAD1-4-mEGFP ft/
ds HA-ftΔCAD1-4
O'
P
Ds-mEGFP
polarity magnitude
DsFL/FtFL
DsΔCAD1-4/FtΔCAD1-4
Ds-mEGFP polarity angle
anterior
posterior

Figure S3. ***In vivo*** **effects of deleting Ds and Ft CAD binding domains. Related to Figure 4.**

(A-D) Adult male wings of the indicated genotypes. Scale bar 25 µm.

(E,F) Quantitation of wing area for the genotypes in Fig.4A-L. Error bars are SD, n=10. Samples were compared to w^{1118} control (black asterisks), $ds\text{-}mEGFP/ds^{UA071}$ (purple asterisks in E) or $HA\text{-}ft/ft^{G\text{-}rv}$ (blue asterisks in F) using ANOVA with Dunnett's multiple comparisons test (*p<0.05, **p<0.01, ****p≤0.0001).

(G,H) Adult wings of $HA\text{-}ft^{\Delta CAD26\text{-}30}/ft^{G\text{-}rv}$ male flies, indicating the two classes of wing shape. Fewer than 10% of emerging flies had class II wings (H), and they emerged late. Scale bar 400 µm.

(I) Quantitation of length-width ratio for $HA\text{-}ft^{\Delta CAD26\text{-}30}/ft^{G\text{-}rv}$ *or* $HA\text{-}ft^{\Delta CAD1\text{-}4\Delta CAD26\text{-}30}/ft^{G\text{-}rv}$ wings, split into class I and class II. Error bars are SD.

(J,K) Adult wings of $ds\text{-}mEGFP\ ft^{G\text{-}rv}/ds^{UA071}\ HA\text{-}ft$ (J) or $ds^{\Delta CAD1\text{-}4}\text{-}mEGFP\ ft^{G\text{-}rv}/ds^{UA071}\ HA\text{-}ft^{\Delta CAD1\text{-}4}$ (K) male flies.

(L,M) Quantitation of length-width ratio (L) and crossvein distance (M) in adult wings of genotypes shown in panels J,K or Fig.4. Values in columns 1-5 are as in Fig.4M-P. Error bars are SD, n=10, except for crossvein distance for $ds^{\Delta CAD1\text{-}4}\text{-}mEGFP\ ft^{G\text{-}rv}/ds^{UA071}\ HA\text{-}ft^{\Delta CAD1\text{-}4}$, where n=8. Where incomplete posterior crossveins were present, crossvein distances were extrapolated. Pre-selected pairs of samples were compared using ANOVA with Šidák's multiple comparisons test (**p≤0.0001).

(N,O) 28 hr APF pupal wings from $ds\text{-}mEGFP\ ft^{G\text{-}rv}/ds^{UA071}\ HA\text{-}ft$ (N) or $ds^{\Delta CAD1\text{-}4}\text{-}mEGFP\ ft^{G\text{-}rv}/ds^{UA071}\ HA\text{-}ft^{\Delta CAD1\text{-}4}$ (O) flies. Images show EGFP fluorescence (green) and HA immunolabelling (magenta). (N', O') Nematics for neighbour vector polarity magnitude, based on EGFP fluorescence. Scale bar 10 µm.

(P) Cell-by-cell polarity measurements based on EGFP fluorescence in the region near the wing margin marked in yellow in N' and O', n=6. Polarity magnitudes were compared using an unpaired t-test (**p≤0.01). Rose plots show the distribution of polarity angles pooled for all wings.

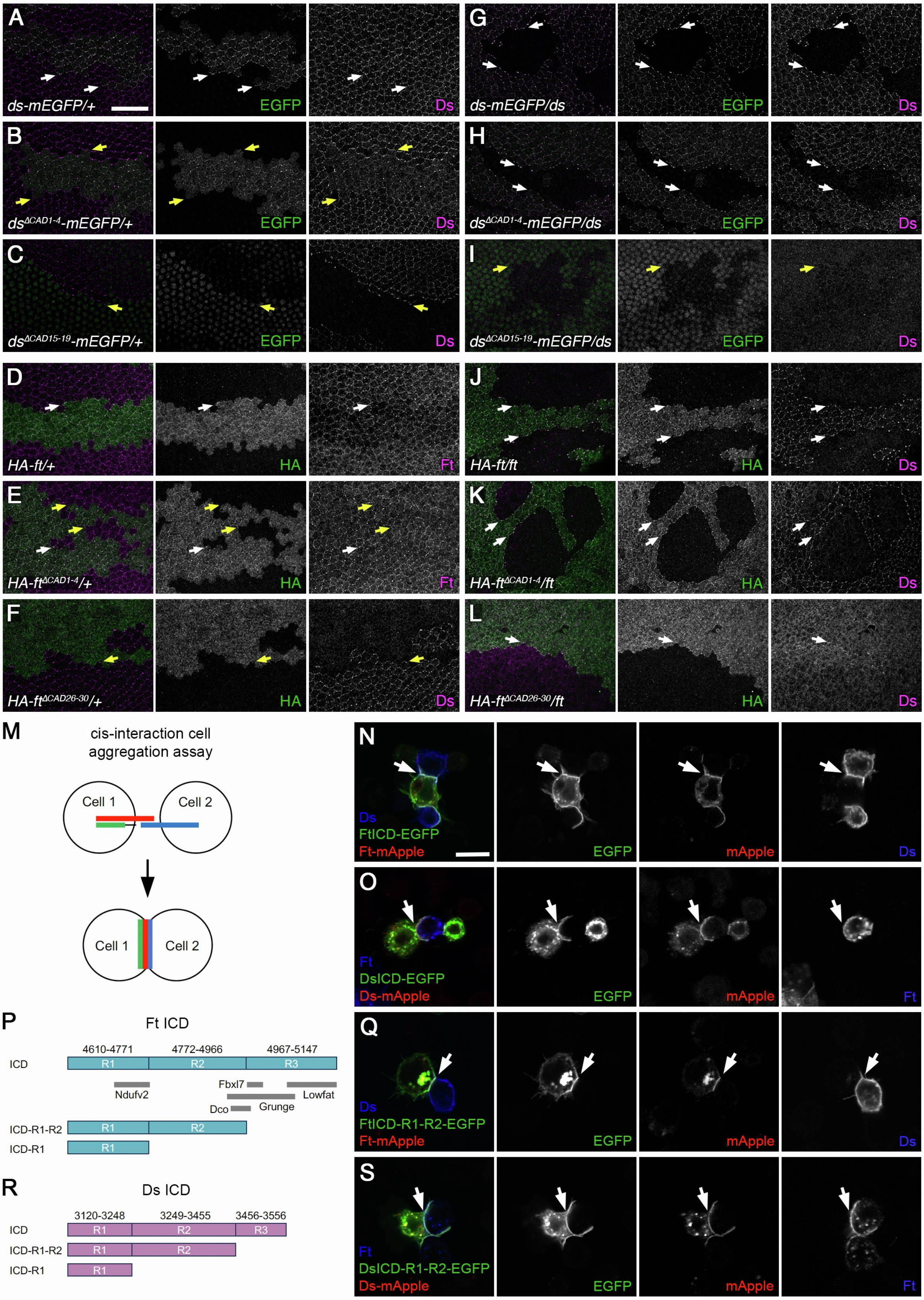
A
ds-mEGFP/+
EGFP
Ds
B
dsΔCAD1-4-mEGFP/+
C
dsΔCAD15-19-mEGFP/+
D
HA-ft/+
HA
Ft
E
HA-ftΔCAD1-4/+
F
HA-ftΔCAD26-30/+
G
ds-mEGFP/ds
H
dsΔCAD1-4-mEGFP/ds
I
dsΔCAD15-19-mEGFP/ds
J
HA-ft/ft
K
HA-ftΔCAD1-4/ft
L
HA-ftΔCAD26-30/ft
M
cis-interaction cell aggregation assay
Cell 1
Cell 2
N
FtICD-EGFP
Ft-mApple
mApple
O
DsICD-EGFP
Ds-mApple
P
Ft ICD
4610-4771
4772-4966
4967-5147
ICD
R1
R2
R3
Ndufv2
Fbxl7
Lowfat
Dco
Grunge
ICD-R1-R2
ICD-R1
Q
FtICD-R1-R2-EGFP
R
Ds ICD
3120-3248
3249-3455
3456-3556
S
DsICD-R1-R2-EGFP

Figure S4. Effects of deleting N-terminal and C-terminal CAD binding sites of Ds and Ft *in vivo*, and cis-interactions. Related to Figure 4.

(A-C) 28 hr APF pupal wings carrying clones of *ds-mEGFP* (A), $ds^{\Delta CAD1\text{-}4}$*-mEGFP* (B) or $ds^{\Delta CAD15\text{-}19}$*-mEGFP* (C) next to wild-type tissue, with EGFP fluorescence (green) and immunolabelled for Ds (magenta). White arrows point to posterior localisation of Ds-mEGFP on clone boundaries (A), while $\mathrm{Ds}^{\Delta \mathrm{CAD1\text{-}4}}$-mEGFP is weakly present at apicolateral cell junctions inside clones but excluded from clone boundaries (yellow arrows in B). $\mathrm{Ds}^{\Delta \mathrm{CAD15\text{-}19}}$-mEGFP is excluded from apicolateral cell junctions both inside clones and at clone boundaries (yellow arrows in C). Scale bar 20 µm.

(D-F) 28 hr APF pupal wings carrying clones of *HA-ft* (D), *HA-*$ft^{\Delta CAD1\text{-}4}$ (E) or *HA-*$ft^{\Delta CAD26\text{-}30}$ (F) next to wild-type tissue, immunolabelled for HA (green) and Ft (D,E) or Ds (F) in magenta. White arrows point to weak anterior localisation of HA-Ft on clone boundaries (D). HA-$\mathrm{Ft}^{\Delta \mathrm{CAD1\text{-}4}}$ is weakly present at apicolateral cell junctions inside clones but excluded from clone boundaries (yellow arrows in E). HA-$\mathrm{Ft}^{\Delta \mathrm{CAD26\text{-}30}}$ is not enriched at apicolateral cell junctions inside clones and is excluded from clone boundaries (yellow arrows in F).

(G-I) 28 hr APF pupal wings carrying clones of *ds-mEGFP* (G), $ds^{\Delta CAD1\text{-}4}$*-mEGFP* (H) or $ds^{\Delta CAD15\text{-}19}$*-mEGFP* (I) next to ds^{UA071} mutant tissue, with EGFP fluorescence (green) and immunolabelled for Ds (magenta). White arrows point to localisation of Ds-mEGFP and $\mathrm{Ds}^{\Delta \mathrm{CAD1\text{-}4}}$-mEGFP to boundaries all around the clone (G,H), while $\mathrm{Ds}^{\Delta \mathrm{CAD15\text{-}19}}$-mEGFP is not at apicolateral cell junctions inside clones or at clone boundaries (yellow arrows in I).

(J-L) 28 hr APF pupal wings carrying clones of *HA-ft* (J), *HA-*$ft^{\Delta CAD1\text{-}4}$ (K) or *HA-*$ft^{\Delta CAD26\text{-}30}$ (L) next to $ft^{G\text{-}rv}$ mutant tissue, immunolabelled for HA (green) and Ds (magenta). White arrows point to localisation of HA-Ft and HA-$\mathrm{Ft}^{\Delta \mathrm{CAD1\text{-}4}}$ to boundaries all around the clone (J,K), and there is weak localisation of HA-$\mathrm{Ft}^{\Delta \mathrm{CAD26\text{-}30}}$ at clone boundaries (white arrows in L). This is consistent with $\mathrm{Ft}^{\Delta \mathrm{CAD26\text{-}30}}$ binding in trans to Ds in neighbouring cells.

(M) Diagram illustrating the S2 cell aggregation assay for cis interactions. Cells are co-transfected with FL Ft-mApple and EGFP-tagged Ft intracellular domain (ICD) fused to the CD2 transmembrane domain, and mixed with cells expressing FL Ds, or vice versa. If cis-interactions occur, Ft-ICD-EGFP is recruited to interfaces where FL Ft-mApple binds to FL Ds.

(N,O,Q,S) Cis-interaction cell aggregation experiments between (N) cells co-expressing FL Ft-mApple and the entire Ft-ICD-EGFP, mixed with cells expressing FL Ds; (O) cells co-expressing FL Ds-mApple and the entire Ds-ICD-EGFP, mixed with cells expressing FL Ft; (Q) cells co-expressing FL Ft-mApple and Ft-ICD-R1-R2-EGFP, mixed with cells expressing FL Ds; (S) cells co-expressing FL Ds-mApple and Ds-ICD-R1-R2-EGFP, mixed with cells

expressing FL Ft. Images show EGFP (green) and mApple (red) fluorescence, and immunolabelled for Ds (N,Q) or Ft (O,S). Arrows point to interfaces between Ds- and Ft-expressing cells. Scale bar 10 μm.

(P,R) Diagram of the Ft-ICD (P) or the Ds-ICD (R), showing the R1, R2 and R3 sub-regions, together with the ICDR-1 and ICDR1-R2 constructs that were tested. Ft ICD-R1 and Ds-ICD-R1 were not recruited to cell interfaces. Also shown are positions of binding sites for known interactors in the Ft ICD [S1-S5].

Table S1. Composition of CAD expression and deletion constructs. Related to STAR Methods.

CAD construct	Amino acids expressed
DsCAD1-2	75-289
DsCAD1-4	75-503
DsCAD1-7	62-841
DsCAD1-15	62-1736
DsCAD1-27	75-3096
DsCAD2-4	170-503
DsCAD3-4	266-503
DsCAD3-7	266-841
DsCAD5-7	495-841
DsCAD5-15	495-1736
DsCAD8-15	800-1736
DsCAD8-21	800-2413
DsCAD10-27	1037-3081
DsCAD14-17	1486-1952
DsCAD15-27	1603-3081
DsCAD16-21	1706-2445
DsCAD17-21	1849-2445
DsCAD17-22	1849-2532
DsCAD17-27	1849-3081
DsCAD18-27	1953-3081
DsCAD19-24	2061-2752
DsCAD19-27	2058-3081
DsCAD22-27	2405-3081
DsCAD25-27	2753-3081
FtCAD1-2	36-291
FtCAD1-3	70-382
FtCAD1-4	36-515
FtCAD1-7	70-820
FtCAD1-15	70-1713
FtCAD1-34	58-3773
FtCAD5-15	495-1713
FtCAD5-25	495-2810

FtCAD2-4	155-515
FtCAD3-4	271-515
FtCAD16-25	1714-2810
FtCAD16-26	1714-2913
FtCAD16-34	1714-3756
FtCAD22-26	2386-2913
FtCAD26	2811-2913
FtCAD26-27	2811-3013
FtCAD26-30	2811-3334
FtCAD26-34	2811-3756
FtCAD27-28	2914-3124
FtCAD27-34	2914-3756
FtCAD29-31	3125-3439
FtCAD31-34	3335-3756
DsCAD1-2-FtCAD3-4	Ds22-233, Ft271-494
FtCAD1-2-FtCAD3-4	Ft36-270, Ds234-451
Ds1-Ft2-Ds3-Ft4	Ds22-121, Ft157-270, Ds234-340, Ft383-494
Ft1-Ds2-Ft3-Ds4	Ft36-156, Ds122-233, Ft271-382, Ds341-451
hDchs1CAD1-4	37-481
hDchs1CAD10-27	995-2939
hFat4CAD1-4	37-481
hFat4CAD26-30	2664-3200
**CAD deletion construct**	**Amino acids deleted**
DsΔCAD1	Δ31-119
DsΔCAD1-2	Δ31-231
DsΔCAD1-4	Δ31-449
DsΔCAD2	Δ143-231
DsΔCAD15-19	Δ1587-2105
DsΔCAD1-4Δ15-19	Δ31-449 and Δ1587-2105
FtΔCAD1	Δ79-154
FtΔCAD1-2	Δ79-268
FtΔCAD1-4	Δ79-492
FtΔCAD26-30	Δ2811-3332
FtΔCAD1-4Δ26-30	Δ79-492 and Δ2811-3332

Table S2. Detailed statistical data. Related to Figures 2, 3 and 4.

**Figure 2H: ANOVA with Dunnett's multiple comparison's test**				
**Sample 1**	**n**	**Sample 2**	**n**	**P value**
DsCAD1-4 next to FtCAD1-4	3	DsCAD1-2 next to FtCAD3-4	3	<0.0001
DsCAD1-4 next to FtCAD1-4	3	FtCAD1-3 next to DsCAD2-4	3	0.0023
DsCAD1-4 next to FtCAD1-4	3	DsCAD1-2-FtCAD3-4	3	0.0004
DsCAD1-4 next to FtCAD1-4	3	FtCAD1-2-DsCAD3-4	3	<0.0001
**Figure 2H: ANOVA with Šídák's multiple comparisons test**				
**Sample 1**	**n**	**Sample 2**	**n**	**P value**
DsCAD1-2-FtCAD3-4	3	FtCAD1-2-DsCAD3-4	3	0.0054
**Figure 3D: ANOVA with Dunnett's multiple comparison's test**				
**Sample 1**	**n**	**Sample 2**	**n**	**P value**
Ds-FL next to Ft-mApple	7	Ds∆CAD1 next to Ft-mApple	5	<0.0001
Ds-FL next to Ft-mApple	7	Ds∆CAD1-2 next to Ft-mApple	5	<0.0001
Ds-FL next to Ft-mApple	7	Ds∆CAD1-4 next to Ft-mApple	5	<0.0001
Ds-FL next to Ft-mApple	7	Ds∆CAD15-19 next to Ft-mApple	5	<0.0001
**Figure 3I: ANOVA with Dunnett's multiple comparison's test**				
**Sample 1**	**n**	**Sample 2**	**n**	**P value**
Ft-FL next to Ds-mApple	5	Ft∆CAD1 next to Ds-mApple	5	0.2629
Ft-FL next to Ds-mApple	5	Ft∆CAD1-2 next to Ds-mApple	6	0.0102
Ft-FL next to Ds-mApple	5	Ft∆CAD1-4 next to Ds-mApple	5	0.0894
Ft-FL next to Ds-mApple	5	Ft∆CAD26-30 next to Ds-mApple	5	0.1452
**Figure 3Q: ANOVA with Šídák's multiple comparisons test**				
**Sample 1**	**n**	**Sample 2**	**n**	**P value**
FtCAD1-4 next to DsCAD1-4	3	FtCAD1-4 next to DsCAD17-22	3	0.0085
FtCAD26-30 next to DsCAD1-4	3	FtCAD26-30 next to DsCAD17-22	3	<0.0001
FtCAD1-4 next to DsCAD1-4	3	FtCAD26-30 next to DsCAD1-4	3	0.0019
FtCAD1-4 next to DsCAD17-22	3	FtCAD26-30 next to DsCAD17-22	3	0.1357
**Figure 4M: ANOVA with Dunnett's multiple comparison's test**				
**Sample 1**	**n**	**Sample 2**	**n**	**P value**
w^{1118}	10	ds^{1}/ds^{UA071}	10	<0.0001
w^{1118}	10	ds^{38K}/ds^{UA071}	10	<0.0001
w^{1118}	10	ds-mEGFP/ds^{UA071}	10	0.0348
w^{1118}	10	$ds^{\Delta CAD1-4}$-mEGFP/ds^{UA071}	10	<0.0001
w^{1118}	10	$ds^{\Delta CAD15-19}$-mEGFP/ds^{UA071}	10	<0.0001
w^{1118}	10	$ds^{\Delta CAD1-4\Delta CAD15-19}$-mEGFP/$ds^{UA071}$	10	<0.0001
ds-mEGFP/ds^{UA071}	10	$ds^{\Delta CAD1-4}$-mEGFP/ds^{UA071}	10	<0.0001
ds-mEGFP/ds^{UA071}	10	$ds^{\Delta CAD15-19}$-mEGFP/ds^{UA071}	10	<0.0001
ds-mEGFP/ds^{UA071}	10	$ds^{\Delta CAD1-4\Delta CAD15-19}$-mEGFP/$ds^{UA071}$	10	<0.0001
**Figure 4M: ANOVA with Tukey's multiple comparison's test**				
**Sample 1**	**n**	**Sample 2**	**n**	**P value**
$ds^{\Delta CAD1-4}$-mEGFP/ds^{UA071}	10	$ds^{\Delta CAD15-19}$-mEGFP/ds^{UA071}	10	<0.0001
$ds^{\Delta CAD1-4}$-mEGFP/ds^{UA071}	10	$ds^{\Delta CAD1-4\Delta CAD15-19}$-mEGFP/$ds^{UA071}$	10	<0.0001
$ds^{\Delta CAD15-19}$-mEGFP/ds^{UA071}	10	$ds^{\Delta CAD1-4\Delta CAD15-19}$-mEGFP/$ds^{UA071}$	10	0.9975
**Figure 4N: ANOVA with Dunnett's multiple comparison's test**				
w^{1118}	10	ds^{1}/ds^{UA071}	10	<0.0001
w^{1118}	10	ds^{38K}/ds^{UA071}	10	-
w^{1118}	10	ds-mEGFP/ds^{UA071}	10	0.004
w^{1118}	10	$ds^{\Delta CAD1-4}$-mEGFP/ds^{UA071}	10	<0.0001
w^{1118}	10	$ds^{\Delta CAD15-19}$-mEGFP/ds^{UA071}	3	<0.0001
w^{1118}	10	$ds^{\Delta CAD1-4\Delta CAD15-19}$-mEGFP/$ds^{UA071}$	0	-
ds-mEGFP/ds^{UA071}	10	$ds^{\Delta CAD1-4}$-mEGFP/ds^{UA071}	10	<0.0001
ds-mEGFP/ds^{UA071}	10	$ds^{\Delta CAD15-19}$-mEGFP/ds^{UA071}	3	<0.0001
ds-mEGFP/ds^{UA071}	10	$ds^{\Delta CAD1-4\Delta CAD15-19}$-mEGFP/$ds^{UA071}$	0	-
**Figure 4N: ANOVA with Tukey's multiple comparison's test**				
**Sample 1**	**n**	**Sample 2**	**n**	**P value**
$ds^{\Delta CAD1-4}$-mEGFP/ds^{UA071}	10	$ds^{\Delta CAD15-19}$-mEGFP/ds^{UA071}	3	<0.0001
$ds^{\Delta CAD1-4}$-mEGFP/ds^{UA071}	10	$ds^{\Delta CAD1-4\Delta CAD15-19}$-mEGFP/$ds^{UA071}$	0	-
$ds^{\Delta CAD15-19}$-mEGFP/ds^{UA071}	3	$ds^{\Delta CAD1-4\Delta CAD15-19}$-mEGFP/$ds^{UA071}$	0	-
**Figure 4O: ANOVA with Dunnett's multiple comparison's test**				
**Sample 1**	**n**	**Sample 2**	**n**	**P value**
w^{1118}	10	ft^{1}/ft^{G-rv}	10	<0.0001

w^{1118}	10	HA-ft/ftG-rv	10	0.0026
w^{1118}	10	HA-ft$^{\Delta CAD1-4}$/ftG-rv	10	<0.0001
w^{1118}	10	HA-ft$^{\Delta CAD26-30}$/ftG-rv	9	<0.0001
w^{1118}	10	HA-ft$^{\Delta CAD1-4 \Delta CAD26-30}$/ftG-rv	10	<0.0001
HA-ft/ftG-rv	10	HA-ft$^{\Delta CAD1-4}$/ftG-rv	10	<0.0001
HA-ft/ftG-rv	10	HA-ft$^{\Delta CAD26-30}$/ftG-rv	9	<0.0001
HA-ft/ftG-rv	10	HA-ft$^{\Delta CAD1-4 \Delta CAD26-30}$/ftG-rv	10	<0.0001
**Figure 4O: ANOVA with Tukey's multiple comparison's test**				
**Sample 1**	**n**	**Sample 2**	**n**	**P value**
HA-ft$^{\Delta CAD1-4}$/ftG-rv	10	HA-ft$^{\Delta CAD26-30}$/ftG-rv	9	0.0699
HA-ft$^{\Delta CAD1-4}$/ftG-rv	10	HA-ft$^{\Delta CAD1-4 \Delta CAD26-30}$/ftG-rv	10	0.0014
HA-ft$^{\Delta CAD26-30}$/ftG-rv	9	HA-ft$^{\Delta CAD1-4 \Delta CAD26-30}$/ftG-rv	10	0.2814
**Figure 4P: ANOVA with Dunnett's multiple comparison's test**				
**Sample 1**	**n**	**Sample 2**	**n**	**P value**
w^{1118}	10	ft^{1}/ft^{G-rv}	10	<0.0001
w^{1118}	10	HA-ft/ftG-rv	10	<0.0001
w^{1118}	10	HA-ft$^{\Delta CAD1-4}$/ftG-rv	10	<0.0001
w^{1118}	10	HA-ft$^{\Delta CAD26-30}$/ftG-rv	9	<0.0001
w^{1118}	10	HA-ft$^{\Delta CAD1-4 \Delta CAD26-30}$/ftG-rv	10	<0.0001
HA-ft/ftG-rv	10	HA-ft$^{\Delta CAD1-4}$/ftG-rv	10	<0.0001
HA-ft/ftG-rv	10	HA-ft$^{\Delta CAD26-30}$/ftG-rv	9	<0.0001
HA-ft/ftG-rv	10	HA-ft$^{\Delta CAD1-4 \Delta CAD26-30}$/ftG-rv	10	<0.0001
**Figure 4P: ANOVA with Tukey's multiple comparison's test**				
**Sample 1**	**n**	**Sample 2**	**n**	**P value**
HA-ft$^{\Delta CAD1-4}$/ftG-rv	10	HA-ft$^{\Delta CAD26-30}$/ftG-rv	9	<0.0001
HA-ft$^{\Delta CAD1-4}$/ftG-rv	10	HA-ft$^{\Delta CAD1-4 \Delta CAD26-30}$/ftG-rv	10	<0.0001
HA-ft$^{\Delta CAD26-30}$/ftG-rv	9	HA-ft$^{\Delta CAD1-4 \Delta CAD26-30}$/ftG-rv	10	0.8811
**Figure 4U: unpaired t-test**				
**Sample 1**	**n**	**Sample 2**	**n**	**P value**
ds-mEGFP/ds^{UA071}	8	ds$^{\Delta CAD1-4}$-mEGFP/ds^{UA071}	9	<0.0001
**Figure 4V: unpaired t-test**				
**Sample 1**	**n**	**Sample 2**	**n**	**P value**
HA-ft/ftG-rv	9	HA-ft$^{\Delta CAD1-4}$/ftG-rv	8	0.1276
**Figure S2H: ANOVA with Dunnett's multiple comparison's test**				
**Sample 1**	**n**	**Sample 2**	**n**	**P value**
DsFL next to Ft-mApple	11	Ds$^{\Delta CAD1-4}$ next to Ft-mApple	7	<0.0001
DsFL next to Ft-mApple	11	Ds$^{\Delta CAD15-19}$ next to Ft-mApple	10	<0.0001
**Figure S2I: ANOVA with Dunnett's multiple comparison's test**				
**Sample 1**	**n**	**Sample 2**	**n**	**P value**
FtFL next to Ds-mApple	10	Ft$^{\Delta CAD1-4}$ next to Ds-mApple	11	0.0045
FtFL next to Ds-mApple	10	Ft$^{\Delta CAD26-30}$ next to Ds-mApple	9	0.154
**Figure S3E: ANOVA with Dunnett's multiple comparison's test**				
**Sample 1**	**n**	**Sample 2**	**n**	**P value**
w^{1118}	10	ds^{1}/ds^{UA071}	10	<0.0001
w^{1118}	10	ds^{38K}/ds^{UA071}	10	0.0100
w^{1118}	10	ds-mEGFP/ds^{UA071}	10	0.3471
w^{1118}	10	ds$^{\Delta CAD1-4}$-mEGFP/ds^{UA071}	10	0.1803
w^{1118}	10	ds$^{\Delta CAD15-19}$-mEGFP/ds^{UA071}	10	0.2578
w^{1118}	10	ds$^{\Delta CAD1-4 \Delta CAD15-19}$-mEGFP/ds^{UA071}	10	0.9983
ds-mEGFP/ds^{UA071}	10	ds$^{\Delta CAD1-4}$-mEGFP/ds^{UA071}	10	0.9628
ds-mEGFP/ds^{UA071}	10	ds$^{\Delta CAD15-19}$-mEGFP/ds^{UA071}	10	0.0015
ds-mEGFP/ds^{UA071}	10	ds$^{\Delta CAD1-4 \Delta CAD15-19}$-mEGFP/ds^{UA071}	10	0.3612
**Figure S3F: ANOVA with Dunnett's multiple comparison's test**				
**Sample 1**	**n**	**Sample 2**	**n**	**P value**
w^{1118}	10	ft^{1}/ft^{G-rv}	10	<0.0001
w^{1118}	10	HA-ft/ftG-rv	10	<0.0001
w^{1118}	10	HA-ft$^{\Delta CAD1-4}$/ftG-rv	10	<0.0001
w^{1118}	10	HA-ft$^{\Delta CAD26-30}$/ftG-rv	9	<0.0001
w^{1118}	10	HA-ft$^{\Delta CAD1-4 \Delta CAD26-30}$/ftG-rv	10	<0.0001
HA-ft/ftG-rv	10	HA-ft$^{\Delta CAD1-4}$/ftG-rv	10	<0.0001
HA-ft/ftG-rv	10	HA-ft$^{\Delta CAD26-30}$/ftG-rv	9	0.0866
HA-ft/ftG-rv	10	HA-ft$^{\Delta CAD1-4 \Delta CAD26-30}$/ftG-rv	10	0.0590
**Figure S3L: ANOVA with Dunnett's multiple comparison's test**				
**Sample 1**	**n**	**Sample 2**	**n**	**P value**

w^{1118}	10	ds-mEGFP/ds^{UA071}	10	0.0010
w^{1118}	10	ds$^{\Delta CAD1-4}$-mEGFP/ds^{UA071}	10	<0.0001
w^{1118}	10	HA-ft/ftG-rv	10	0.0003
w^{1118}	10	HA-ft$^{\Delta CAD1-4}$/ftG-rv	10	<0.0001
w^{1118}	10	ds-mEGFP ft^{G-rv}/ds^{UA071} HA-ft	10	0.1835
w^{1118}	10	ds$^{\Delta CAD1-4}$-mEGFP ft^{G-rv}/ds^{UA071} HA-ft$^{\Delta CAD1-4}$	10	<0.0001
**Figure S3L: ANOVA with Tukey's multiple comparison's test**				
**Sample 1**	**n**	**Sample 2**	**n**	**P value**
ds-mEGFP/ds^{UA071}	10	ds$^{\Delta CAD1-4}$-mEGFP/ds^{UA071}	10	<0.0001
HA-ft/ftG-rv	10	HA-ft$^{\Delta CAD1-4}$/ftG-rv	10	<0.0001
ds-mEGFP ft^{G-rv}/ds^{UA071} HA-ft	10	ds$^{\Delta CAD1-4}$-mEGFP ft^{G-rv}/ds^{UA071} HA-ft$^{\Delta CAD1-4}$	10	<0.0001
ds$^{\Delta CAD1-4}$-mEGFP/ds^{UA071}	10	HA-ft$^{\Delta CAD1-4}$/ftG-rv	10	0.0025
ds$^{\Delta CAD1-4}$-mEGFP/ds^{UA071}	10	ds$^{\Delta CAD1-4}$-mEGFP ft^{G-rv}/ds^{UA071} HA-ft$^{\Delta CAD1-4}$	10	0.0058
HA-ft$^{\Delta CAD1-4}$/ftG-rv	10	ds$^{\Delta CAD1-4}$-mEGFP ft^{G-rv}/ds^{UA071} HA-ft$^{\Delta CAD1-4}$	10	>0.9999
**Figure S3M: ANOVA with Dunnett's multiple comparison's test**				
**Sample 1**	**n**	**Sample 2**	**n**	**P value**
w^{1118}	10	ds-mEGFP/ds^{UA071}	10	0.0033
w^{1118}	10	ds$^{\Delta CAD1-4}$-mEGFP/ds^{UA071}	10	<0.0001
w^{1118}	10	HA-ft/ftG-rv	10	<0.0001
w^{1118}	10	HA-ft$^{\Delta CAD1-4}$/ftG-rv	10	<0.0001
w^{1118}	10	ds-mEGFP ft^{G-rv}/ds^{UA071} HA-ft	10	0.0046
w^{1118}	10	ds$^{\Delta CAD1-4}$-mEGFP ft^{G-rv}/ds^{UA071} HA-ft$^{\Delta CAD1-4}$	8	<0.0001
**Figure S3M: ANOVA with Tukey's multiple comparison's test**				
**Sample 1**	**n**	**Sample 2**	**n**	**P value**
ds-mEGFP/ds^{UA071}	10	ds$^{\Delta CAD1-4}$-mEGFP/ds^{UA071}	10	<0.0001
HA-ft/ftG-rv	10	HA-ft$^{\Delta CAD1-4}$/ftG-rv	10	<0.0001
ds-mEGFP ft^{G-rv}/ds^{UA071} HA-ft	10	ds$^{\Delta CAD1-4}$-mEGFP ft^{G-rv}/ds^{UA071} HA-ft$^{\Delta CAD1-4}$	8	<0.0001
ds$^{\Delta CAD1-4}$-mEGFP/ds^{UA071}	10	HA-ft$^{\Delta CAD1-4}$/ftG-rv	10	0.0269
ds$^{\Delta CAD1-4}$-mEGFP/ds^{UA071}	10	ds$^{\Delta CAD1-4}$-mEGFP ft^{G-rv}/ds^{UA071} HA-ft$^{\Delta CAD1-4}$	8	0.0977
HA-ft$^{\Delta CAD1-4}$/ftG-rv	10	ds$^{\Delta CAD1-4}$-mEGFP ft^{G-rv}/ds^{UA071} HA-ft$^{\Delta CAD1-4}$	8	>0.9999
**Figure S3P: unpaired t-test**				
**Sample 1**	**n**	**Sample 2**	**n**	**P value**
ds-mEGFP ft^{G-rv}/ds^{UA071} HA-ft	6	ds$^{\Delta CAD1-4}$-mEGFP ft^{G-rv}/ds^{UA071} HA-ft$^{\Delta CAD1-4}$	6	0.0053

Supplemental references

S1. Fanto, M., Clayton, L., Meredith, J., Hardiman, K., Charroux, B., Kerridge, S., and McNeill, H. (2003). The tumor-suppressor and cell adhesion molecule Fat controls planar polarity via physical interactions with Atrophin, a transcriptional co-repressor. Development *130*, 763-774. 10.1242/dev.00304.

S2. Mao, Y., Kucuk, B., and Irvine, K.D. (2009). *Drosophila lowfat*, a novel modulator of Fat signaling. Development *136*, 3223-3233. 10.1242/dev.036152.

S3. Sopko, R., Silva, E., Clayton, L., Gardano, L., Barrios-Rodiles, M., Wrana, J., Varelas, X., Arbouzova, N., Shaw, S., Saburi, S., et al. (2009). Phosphorylation of the tumor suppressor Fat Is regulated by Its ligand Dachsous and the kinase Discs Overgrown. Curr. Biol. *19*, 1112-1117. 10.1016/j.cub.2009.05.049.

S4. Bosch, J.A., Sumabat, T.M., Hafezi, Y., Pellock, B.J., Gandhi, K.D., and Hariharan, I.K. (2014). The *Drosophila* F-box protein Fbxl7 binds to the protocadherin Fat and regulates Dachs localization and Hippo signaling. Elife *3*, e03383. 10.7554/eLife.03383.

S5. Sing, A., Tsatskis, Y., Fabian, L., Hester, I., Rosenfeld, R., Serricchio, M., Yau, N., Bietenhader, M., Shanbhag, R., Jurisicova, A., et al. (2014). The atypical cadherin Fat directly regulates mitochondrial function and metabolic state. Cell *158*, 1293-1308. 10.1016/j.cell.2014.07.036.
